# Targeting SPINK1 in the damaged tumour microenvironment alleviates therapeutic resistance

**DOI:** 10.1038/s41467-018-06860-4

**Published:** 2018-10-17

**Authors:** Fei Chen, Qilai Long, Da Fu, Dexiang Zhu, Yan Ji, Liu Han, Boyi Zhang, Qixia Xu, Bingjie Liu, Yan Li, Shanshan Wu, Chen Yang, Min Qian, Jianmin Xu, Suling Liu, Liu Cao, Y. Eugene Chin, Eric W.-F. Lam, Jean-Philippe Coppé, Yu Sun

**Affiliations:** 1 Key Laboratory of Tissue Microenvironment and Tumour, Shanghai Institutes for Biological Sciences, University of Chinese Academy of Sciences, Chinese Academy of Sciences, Shanghai, 200031 China; 2 Department of Urology, Zhongshan Hospital, Fudan University, Shanghai, 200032 China; 3 Central Laboratory for Medical Research, Shanghai Tenth People’s Hospital, Tongji University School of Medicine, Shanghai, 200072 China; 4 Department of General Surgery, Zhongshan Hospital, Fudan University, Shanghai, 200032 China; 5 Institute of Health Sciences, Shanghai Jiao Tong University School of Medicine & Shanghai Institutes for Biological Sciences, Chinese Academy of Sciences, Shanghai, 200031 China; 6 Fudan University Shanghai Cancer Center & Institutes of Biomedical Sciences, Shanghai Medical College, Key Laboratory of Breast Cancer in Shanghai, Innovation Center for Cell Signaling Network, Cancer Institutes, Fudan University, Shanghai, 200032 China; 7 Key Laboratory of Medical Cell Biology, China Medical University, Shenyang, 110122 China; 8 Institute of Biology and Medical Sciences, Soochow University Medical College, 199 Renai Road, Suzhou, 215123 Jiangsu China; 9 Department of Surgery and Cancer, Imperial College London, London, W12 0NN UK; 10 Department of Laboratory Medicine, Helen Diller Family Comprehensive Cancer Center, University of California, San Francisco, CA 94115 USA; 11 Department of Medicine, VAPSHCS, University of Washington, Seattle, WA 98195 USA

## Abstract

Chemotherapy and radiation not only trigger cancer cell apoptosis but also damage stromal cells in the tumour microenvironment (TME), inducing a senescence-associated secretory phenotype (SASP) characterized by chronic secretion of diverse soluble factors. Here we report serine protease inhibitor Kazal type I (SPINK1), a SASP factor produced in human stromal cells after genotoxic treatment. DNA damage causes SPINK1 expression by engaging NF-κB and C/EBP, while paracrine SPINK1 promotes cancer cell aggressiveness particularly chemoresistance. Strikingly, SPINK1 reprograms the expression profile of cancer cells, causing prominent epithelial-endothelial transition (EET), a phenotypic switch mediated by EGFR signaling but hitherto rarely reported for a SASP factor. In vivo, SPINK1 is expressed in the stroma of solid tumours and is routinely detectable in peripheral blood of cancer patients after chemotherapy. Our study substantiates SPINK1 as both a targetable SASP factor and a novel noninvasive biomarker of therapeutically damaged TME for disease control and clinical surveillance.

## Abstract (web summary)

Tumour microenvironment actively contributes to drug resistance in clinical oncology. Here, the authors show that genotoxic stress induces senescence in human stromal cells, which in turn secrete serine protease inhibitor Kazal type 1 (SPINK1) and promote acquired resistance of cancer cells via EGFR-mediated paracrine signaling.

## Introduction

Tumour development involves the co-evolution of transformed cells and nearby stroma^1^. Numerous studies have demonstrated that the tumour microenvironment (TME) plays critical roles in disease progression, including but not limited to the generation of profound impacts on therapeutic efficacy^2^. In contrast to cancer cell intrinsic resistance, which is associated with preexisting genetic and/or epigenetic alterations, acquired resistance arises upon drug treatment. Specifically, tumour resistance driven by the pathologically active host stroma has attracted substantial attention in recent years^3–5^. As mutations rarely occur in the stroma, understanding and managing the TME-mediated resistance can presumably advance the development of innovative therapeutic strategies^1^. With increasing arsenal of anticancer agents, it is likely that treatment resistance can be more effectively circumvented through patient stratification based on predictive biomarkers and rational design of drug combinations to target both cancer cells and the surrounding TME^6^.

Although most clinical regimens debulk tumours through clearance of the rapidly expanding malignant cells, their off-target effects frequently trigger irreparable damage in benign stromal cells and cause typical cellular senescence, a process accompanied by the appearance of a senescence-associated secretory phenotype (SASP)^7^. The SASP can facilitate tissue homeostasis by enhancing wound healing, tissue repair, and recruitment of immune cells to eliminate damaged cells^8^, however, more studies support the implication of the SASP in age-related pathologies^9,10^. Importantly, we and others have reported that secretion of a myriad of soluble factors including cytokines, chemokines, and growth factors produced by the SASP, can promote chemoresistance of the residual cancer cells that survival early treatment^11–13^. While the SASP is entering the spotlight of intensive research in a global scope, it remains unclear whether specific components of the full SASP spectrum can intensively drive cancer resistance in treatment conditions. Further, exploration of the functional mechanisms that regulate the expression of major SASP effectors, and development of therapeutic strategies to restrain deleterious consequences of the SASP, represent intriguing but challenging issues. Although reactive stroma is defined as a pathologically dynamic entity in tumour progression^14^, the relevance of a SASP-manifesting “senescent stroma” to malignancy development and histopathologic features/markers of stromal cells in transition from a naive to the senescence state remain less documented.

Among diverse soluble factors released by human stromal cells developing the SASP after genotoxic stress, we noticed SPINK1, a serine peptidase inhibitor Kazal type 1, which emerged in the high ranking SASP expression list^12^. Despite the presence of a subset of SASP components that are enzymes per se, such as members of the matrix metalloproteinase (MMP) family, the emergence of enzymatic inhibitors including TIMP2^7^ and SPINK1^12^ suggest the complexity of the SASP and the pathological impact it may exert on disease progression. Originally purified from the urine of an ovarian cancer patient^15^, SPINK1 is also known as pancreatic secretory trypsin inhibitor (PSTI) or tumour-associated trypsin inhibitor (TATI), and prevents premature activation of proteases in the pancreas^16^. Beyond basal expression in pancreatic acinar cells, SPINK1 is diagnosed in multiple human cancer types and correlated with adverse clinical outcomes^17^. However, the mechanism underlying the treatment-induced expression of SPINK1 in human stroma and its pathological implications remain poorly defined. In this study, we elucidated several fundamental but hitherto-unknown aspects of stromal SPINK1 in treatment settings and investigated its correlation with therapeutic resistance. Our findings establish SPINK1 as both a tumour-promoting factor that is targetable to prevent disease exacerbation and an important biomarker to monitor the TME response to anticancer agents in clinical settings.

## Results

### DNA damage induces SPINK1 expression in human stromal cells

**Fig. 1:**
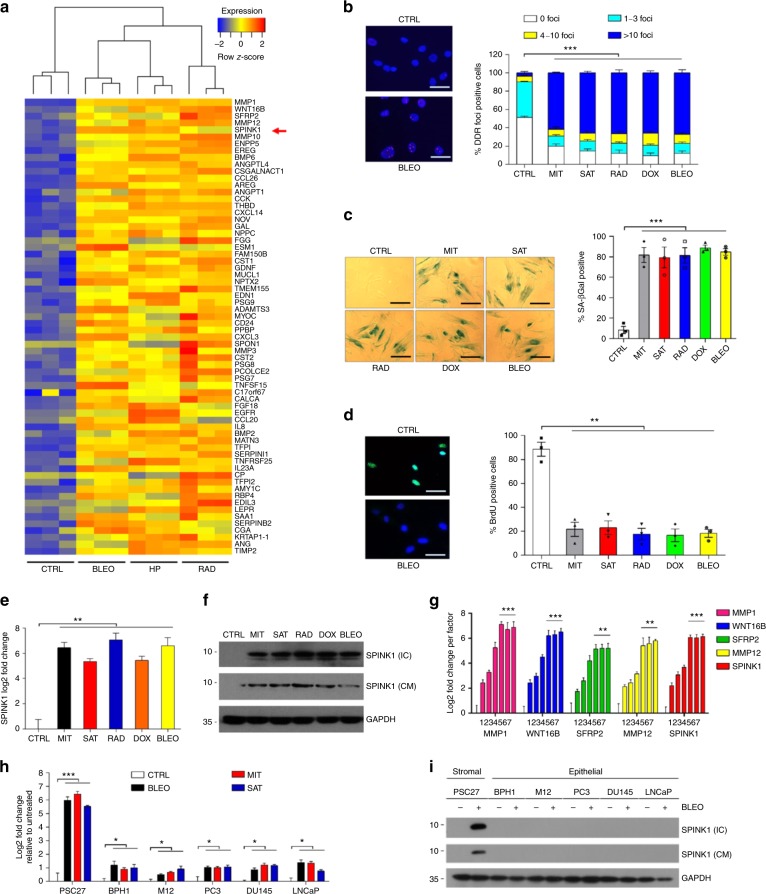
DNA damage to human stromal cells induces expression of secreted factors including SPINK1. **a** Transcriptome-wide profiling of gene expression changes in the primary normal human prostate stromal cell line (PSC27). Cell lysates were collected for analysis 7 days after treatment. CTRL control, BLEO bleomycin, HP hydrogen peroxide, RAD radiation. Red arrow, SPINK1. Agilent microarray data were adapted from Sun et al. with permission from Nature Medicine, copyright 2012. Genes with upregulation fold change >3.0 are listed. **b** Representative immunofluorescence staining images (γH2AX, left) and comparative statistics (right) of DNA damage foci (DDR) in PSC27 cells treated by MIT (mitoxantrone), SAT (satraplatin), RAD (radiation), DOX (doxorubicin), BLEO (bleomycin). DDR were classified into 4 sub-categories including 0, 1–3, 4–10, and >10 foci per cell. Scale bars, 15 μm. **c** SA-β-Gal staining of PSC27 cells 7 days after treatment by various agents used in (**b**). Scale bars, 15 μm. **d** BrdU staining of stromal cells treated by different agents used in (**b**). Scale bars, 20 μm. **e** Quantitative RT-PCR assay of SPINK1 expression 7 days after treatment by various agents. Signals normalized to CTRL (untreated). **f** Immunoblot analysis of SPINK1 expression in stromal cells 7 days after treatment as applied in (**e**). IC intracellular samples, CM conditioned media. GAPDH, loading control. **g** Time course expression assessment of a subset of typical SASP factors after treatment of stromal cells. *X*-axis, 1, 2, 3, 4, 5, 6, and 7 refer to experimental timepoints of 0, 1, 3, 5, 7, 10, and 15 day(s) post-treatment, respectively. Gene primers are listed in Supplementary Table 4. **h** Comparative appraisal of SPINK1 transcription in stromal cells (PSC27) versus transformed or neoplastic epithelial cells (BPH1, M12, PC3, DU145, and LNCaP). Signals normalized to CTRL (untreated sample) per cell line. **i** SPINK1 protein expression in stromal and epithelial cells after BLEO treatment. IC intracellular extracts, CM conditioned media. GAPDH, loading control. Data are shown as mean ± SD and representative of 3 independent experiments. *P* values were calculated by one-way (**c**, **d**, **e**, **g**, **h**) and two-way (**b**) ANOVA (^*P* > 0.05; *P < 0.05; **P < 0.01; ***P < 0.001)

Many studies reported that SPINK1 is frequently mutated or overexpressed in human bladder, colon, liver, and prostate malignancies^18–21^. However, the vast majority of SPINK1-associated cancer research has been focused on neoplastic cells per se, leaving the host-resident stroma largely overlooked. We recently noticed that a prostate stromal cell line PSC27, comprising predominantly fibroblasts but with a minor percentage of other stroma cell lineages including endothelial and immune cells of the TME, produces a large number of SASP factors after exposure to cytotoxic insults particularly those generated by genotoxic chemotherapy or ionizing radiation^12^. Notably, SPINK1 emerged as one of the major SASP factors as formerly revealed by microarray (Fig. 1a)^12^. To consolidate the finding and expand the study, we employed a subset of DNA damaging agents including mitoxantrone (MIT), satraplatin (SAT), γ-radiation (RAD), doxorubicin (DOX), and bleomycin (BLEO) to treat stromal cells. In vitro examination indicated that cells displayed substantially enhanced number of DNA damage foci (γH2AX), increased lysosomal activity (SA-β-Gal), and reduced DNA synthesis (Fig. 1b–d), indicative of typical cell cycle arrest companied by cellular senescence. Subsequent examination at both mRNA and protein levels confirmed an inducible expression nature of SPINK1 in response to multiple genotoxic agents (*P* < 0.01 at transcript level) (Fig. 1e, f). Interestingly, the expression pattern of SPINK1 resembled that of other hallmark SASP factors including MMP1, WNT16B, SFRP2, and MMP12, which is characterized by a gradual increment until cells entered a platform within 7–8 days after treatment (*P* < 0.01 for SFRP2 and MMP12, *P* < 0.001 for others) (Fig. 1g).

Upon assessment of SPINK1 expression as a SASP factor among several cell lines of prostate origin, we found stromal cells significantly more inducible for SPINK1 than epithelial cells, implying a special mechanism that supports SPINK1 production in prostate stromal cells under genotoxic pressure (*P* < 0.001 for stromal, *P* < 0.05 for epithelial) (Fig. 1h, i). The stroma–epithelium differential expression pattern was subsequently confirmed in a group of cell lines of human breast origin, including a stromal line HBF1203 and several cancer cell lines regardless of their malignancy properties, suggesting an organ- or tissue type-independent nature of SPINK1 induction (Supplementary Fig. 1a–f).

Further, we asked if SPINK1 expression per se is responsible for cellular senescence and associated events. To address this, we evaluated the phenotypes of human stromal cells lentivirally transduced with a construct to overexpress SPINK1 (PSC27^SPINK1^ and HBF1203^SPINK1^, respectively), but found no significant alterations in DNA damage response (DDR), lysosomal activity, DNA synthesis rate, and proliferation potential of these cells, regardless of their organ origin (Supplementary Fig. 1g–l). Specifically, production of extracellular proteins such as epidermal growth factor (EGF), a structural homolog of SPINK1, remained unchanged (Supplementary Fig. 1g). Therefore, SPINK1 upregulation is indeed part of the biological consequence, rather than a stimulating cause of cellular senescence.

### Stromal SPINK1 is correlated with poor clinical outcome

**Fig. 2:**
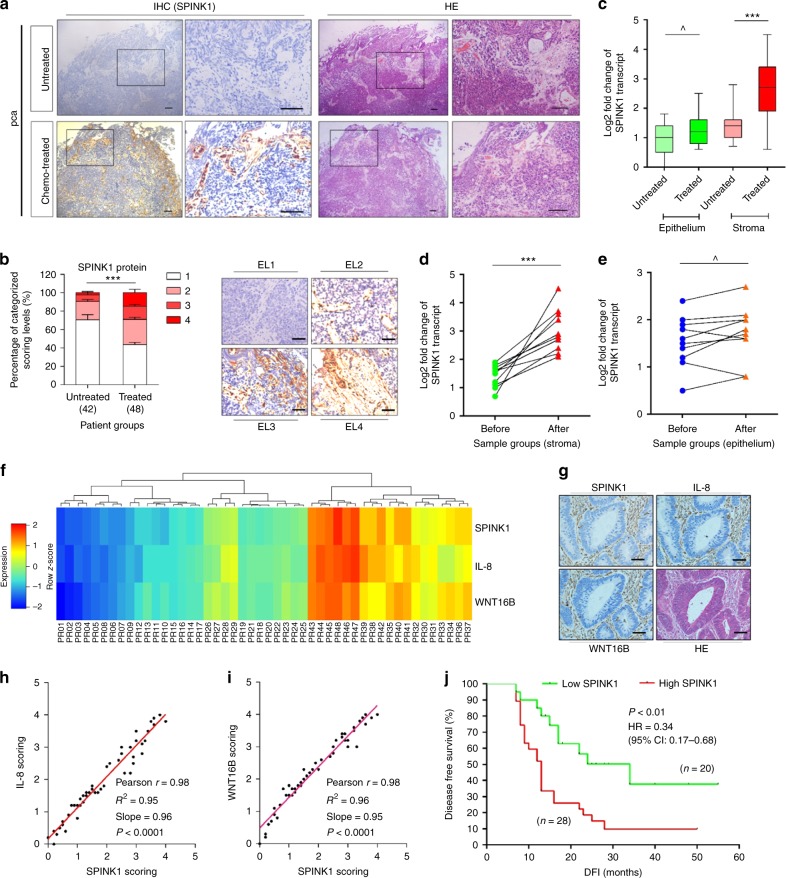
SPINK1 expression in human prostate stroma after chemotherapy is correlated with poor clinical survival. **a** Histochemical images of SPINK1 expression in human prostate cancer (PCa) tissues. Rectangular regions in left images zoomed on the right, all samples acquired from the same patient. Scale bars, 100 μm. **b** Pathological assessment of stromal SPINK1 expression in PCa samples (42 versus 48). Scoring categories: 1, negative; 2, weak; 3, moderate; 4, strong expression. Left, statistical comparison. Right, representative images. ES expression level. Scale bars, 50 µm. **c** Boxplot summary of SPINK1 transcript expression in tumour and stroma. Signals normalized to the lowest value in the untreated epithelium group. Samples from 10 patients out of untreated and treated groups were randomly selected. **d** Comparative analysis of SPINK1 expression at transcription level between stromal cells collected before and after chemotherapy. Each dot represents an individual patient, with the data of “before” and “after” connected to allow direct assessment of SPINK1 induction in the same individual patient. **e** Comparative analysis of SPINK1 expression at transcription level in epithelial cells collected from the same individual patients as described in (**d**). **f** Pathological correlation between SPINK1, IL-8, and WNT16B in the stroma of 48 PCa patients after treatment. Columns represent individual patients, rows different SASP factors. Scores of each patient averaged from 3 independent pathological readings. **g** Representative images of SPINK1, IL-8, and WNT16B expression in the TME of post-treatment patients. Scale bars, 100 μm. **h** Statistical correlation between SPINK1 and IL-38 scores in the 48 tumours with matching protein expression data. **i** Statistical correlation between SPINK1 and WNT16B scores in the same tumours as described in (**h**). **j** Kaplan–Meier analysis. Disease-free survival (DFS) stratified according to SPINK1 expression. DFS represents the length (months) of period calculated from the date of treatment completion to the point of first time disease relapse. Data in plots are shown as mean ± SD and representative of 3 biological replicates. *P* values were calculated by Student’s *t*-test (**c**–**e**), one-way ANOVA (**b**), and log-rank (Mantel–Cox) test (**j**) (^*P* > 0.05; ***P < 0.001). HR hazard ratio

Experimental data from in vitro treatments prompted us to further determine whether SPINK1 is produced by the TME, a pathological niche that comprises multiple stromal cell subpopulations. We investigated the biospecimens of a cohort of prostate cancer (PCa) patients who developed primary tumours and underwent chemotherapy involving genotoxic agents. Surprisingly, SPINK1 was significantly expressed in the prostate tissues of patients after chemotherapy, but not before (Fig. 2a). In line with our in vitro data, upregulated SPINK1 was essentially localized in the stroma, in sharp contrast to the surrounding epithelium which had limited or no staining (Fig. 2a).

Enhanced SPINK1 expression in patient tissues post- versus pre-chemotherapy was also consolidated by a predetermined pathological assessment procedure that allowed quantitative appraisal of a target protein expression according to its immunohistochemistry (IHC) staining intensity (*P* < 0.001) (Fig. 2b). Transcript assays upon laser capture microdissection (LCM) of cell lineage from the primary tissues indicated SPINK1 more readily induced in the stromal rather than epithelial cell populations (*P* < 0.001 versus *P* > 0.05) (Fig. 2c). To substantiate the in vivo inducibility of SPINK1, we analyzed a subset of patients whose pre- and post-chemotherapy biospecimens were both available, and found overtly upregulated SPINK1 in the stroma, but not epithelium, of each individual after chemotherapy (Fig. 2d, e). Further, we noticed that SPINK expression change pattern in the damaged TME is largely concurrent with that of IL-8 and WNT16B, two canonical SASP factors of human stroma cells (Fig. 2f, g)^12^. The correlation between SPINK1 and IL-8 or WNT16B expression in the damaged TME was further confirmed by pathological assessment of their expression in the post-treatment patients (Fig. 2h, i). More importantly, Kaplan–Meier analysis of PCa patients stratified according to SPINK1 expression in their tumour stromal compartments suggested a significant but negative correlation between stromally expressed SPINK1 and disease-free survival (DFS) of treated patients (*P* < 0.01, log-rank test) (Fig. 2j).

As supporting evidence, the distinct pathological properties of SPINK1 were reproduced by extended studies that recruited individual cohorts of human breast cancer (BCa) and colorectal cancer (CRC) patients (*P* < 0.01 for BCa and *P* < 0.05 for CRC cohorts by log-rank test, respectively) (Supplementary Fig. 2a–l). Of note, Cox proportional hazard regression analyses of these patients indicated a significant correlation of stromal SPINK1 with poor cancer survival (Supplementary Tables 1–3). Thus, our data suggested that SPINK1 expression level in tumour stroma can act as an SASP-associated independent predictor of prognosis, which is exploitable in stratifying the risk of disease relapse and clinical mortality in post-treatment patients, and that SPINK1 production by stroma may have a causal role in tumour progression.

### SPINK1 expression is mainly mediated by the NF-κB complex

**Fig. 3:**
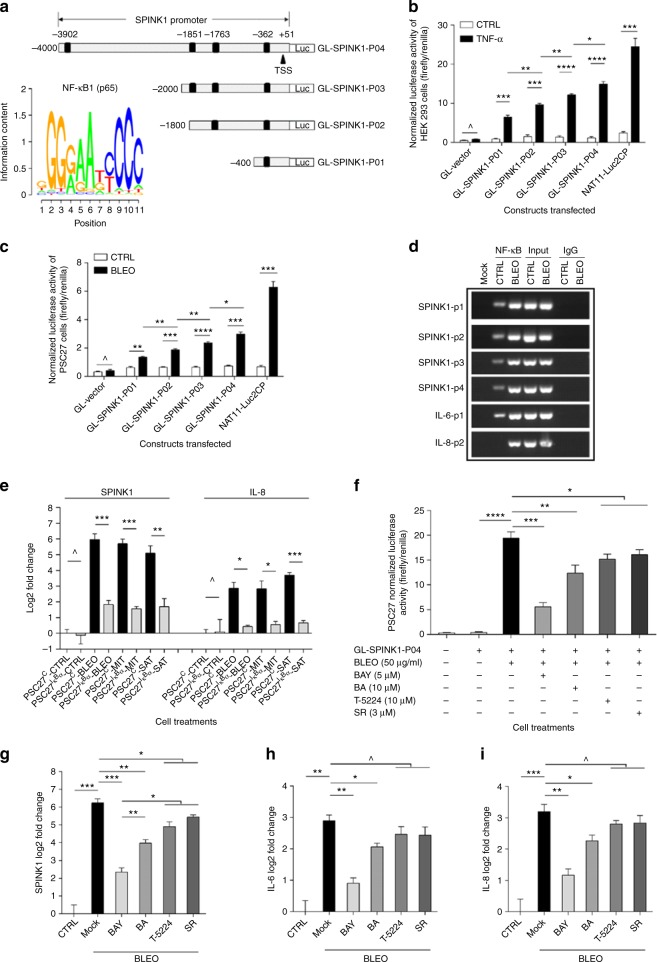
DNA damage induces SPINK1 expression in stromal cells via NF-κB and C/EBP. **a** Schematic of putative NF-κB binding sites in the proximal region of SPINK1 promoter. Reporter constructs was generated by sequential cloning of promoter fragments into a pGL4.22 vector (pGL-SPINK1-P01 to P04) encoding firefly luciferase. TSS transcription start site. Lower-left inlet, consensus binding motif of the NF-κB subunit p65. **b** Luciferase activity assessment upon exposure of 293T cells pre-transfected with the individual SPINK1 promoter constructs to TNF-α at 20 ng/ml in culture. NAT11-Luc2CP, a construct encoding multiple copies of typical NF-κB binding sequences and an optimized IL-2 minimal promoter served as a positive control. **c** Luciferase activity assay with lysates of PSC27 cells pre-transfected with each of the constructs used in (**b**) prior to treatment by 50 μg/ml bleomycin (BLEO). **d** ChIP assay to identify potential NF-κB binding sites in the proximal promoter of SPINK1. Left, SPINK1-p1/p2/p3/p4 denotes 4 representative genomic sites in SPINK1 promoter region, selective NF-κB binding sites from IL-6 and IL-8 served as positive controls. **e** SPINK1 and IL-8 transcript expression in PSC27 cells stably expressing an NF-κB-null mutant and treated by BLEO, MIT, or SAT. **f** Construct pGL-SPINK1-P04 was transiently transfected into PSC27 cells before BLEO treatment. BAY (Bay 11-7982, 5 μM), BA (betulinic acid, 10 μM), T-5224 (10 μM) were applied as small molecule inhibitors against NF-κB, C/EBP family, and AP-1. SR (SR 11302, 3 μM) served as a positive control against AP-1. **g** PSC27 cells were treated in the same conditions as in (**f**), and subject to qRT-PCR analysis. SPINK1 expression was compared between CTRL (untreated), Mock (PBS-treated), BAY, BA, T-5224, and SR treatment groups. **h** Expression of IL-6 transcript in PSC27 cells treated as in (**g**). **i** Expression of IL-8 transcript in PSC27 cells treated as in (**g**). Data are mean ± SD and representative of 3 independent experiments. *P* values were calculated by Student’s *t*-test (**b**, **c**, **e**–**i**) (^*P* > 0.05; *P < 0.05; **P < 0.01; ***P < 0.001; ****P < 0.0001)

Given the remarkable induction of SPINK1 in stromal cells after in vitro and in vivo genotoxic insults, we interrogated the mechanism functionally underlying SPINK1 expression. As one of the major transcriptional machineries in mammalian cells, the NF-κB complex is responsible for the expression of multiple SASP factors in the case of oncogene- or therapy-induced senescence (OIS or TIS)^22,23^. We therefore sought to determine whether DNA damage-induced SPINK1 expression is mediated by NF-κB. Bioinformatics analysis revealed several NF-κB binding motifs in human *SPINK1* promoter region approximately 4000 bp upstream of the transcription starting site (Fig. 3a). Subsequent reporter assays confirmed the functional involvement of these NF-κB binding motifs using a group of *SPINK1* promoter constructs. Compared to untreated sample of 293T or PSC27 cells, both tumour necrosis factor α (TNF-α), a potent NF-κB activator, and the genotoxic agent BLEO remarkably enhanced SPINK1 reporter activity, respectively (Fig. 3b, c). The results were further consolidated by the treatment of these cells with an NF-κB stimulator IL-1α or SAT, respectively (Supplementary Fig. 3a–b). ChIP-PCR assays indicated that each of these binding sites was indeed a bona fide motif bound by NF-κB after DNA damage (Fig. 3d). Functional involvement of NF-κB was confirmed by a PSC27 subline previously established to stably express a mutant IκBα (PSC27^IκBα^), which prevents IκB kinase (IKK)-dependent degradation of IκBα thus attenuates NF-κB signaling^12^. We found PSC27^IκBα^ cells with disabled NF-κB exhibited markedly reduced SPINK1 transcription, regardless of the genotoxic agents used for the in vitro expression assay (Fig. 3e).

In addition to NF-κB, other transcription factors including C/EBP and AP-1 family members were also reported to be associated with SASP expression^24,25^, whereas their functional relevance in SPINK1 induction remains unexplored. To this end, we used betulinic acid (BA), a pentacyclic triterpenoid that inhibits the C/EBP family^26^, and T-5224, a selective suppressor of AP-1 molecules^27^, to treat PSC27 cells pre-transfected with a vector encoding the approximal *SPINK1* promoter upstream of a luciferase transgene reporter. DNA damage induced by genotoxic agents triggered a pronounced increase of luciferase signal, which was essentially abolished by Bay 11-7082, a potent NF-κB inhibitor^28^ (*P* < 0.001 for both BLEO and SAT-damaged cells) (Fig. 3f and Supplementary Fig. 3c). Although treatment with either BA or T-5224 resulted in a dramatic decrease of the reporter signal, their reduction extents were generally less than that caused by Bay 11-7082 (BAY) (*P* < 0.01 for BA and *P* < 0.05 for T-5224, respectively) (Fig. 3f). Further, transcript analysis indicated that DNA damage-induced SPINK1 expression was most effectively restrained by NF-κB inhibition, not C/EBP or AP-1 blockade, although all factors appeared to be involved (*P* < 0.001 for BAY, *P* < 0.01 for BA, *P* < 0.05 for T-5224) (Fig. 3g). The case of SPINK1 expression partially resembled those of IL-6 and IL-8, two SASP hallmark factors, expression of which seems to be mediated by NF-κB and C/EBP but independent of AP-1 (Fig. 3h, i). Together, these data suggest that NF-κB plays a predominant role in mediating stromal SPINK1 expression in genotoxic settings, although other transcription factors are also functionally operative (Supplementary Fig. 3d).

### Paracrine SPINK1 alters recipient cancer cell phenotypes

Previous studies reported SPINK1 as an autocrine factor in promoting malignant phenotypes of PCa cells, including enhanced invasion and proliferation^29^. Here, we investigated the effect of paracrine SPINK1 on PCa cells via culturing with conditioned media (CM) of stroma cells which upon SPINK1 knockdown exhibited neither nonspecific expression changes of other soluble factors such as EGF nor development of cellular senescence (Supplementary Fig. 4a). Upon treatment with the CM from PSC27 cells overexpressing SPINK1 (PSC27^SPINK1^), we observed significantly increased proliferation of several PCa cell lines including PC3, DU145, LNCaP, and M12 (*P* < 0.01) (Supplementary Fig. 4b), accompanied by enhanced migration and invasion (Supplementary Fig. 4c–d). However, these effects were almost completely eliminated by SPINK1-specific shRNAs (Supplementary Fig. 4a), which retained normal proliferative potential of stromal cells but resulted in essentially reversed phenotypes of recipient cancer cells (Supplementary Fig. 4b–d). More importantly, SPINK1 enhanced the resistance of PCa cells to MIT, a DNA-targeting chemotherapeutic agent frequently used for human malignancies including PCa^30,31^ (Supplementary Fig. 4e). MIT induced cleavage of caspase 3, a process that was remarkably weakened by SPINK1 but can be maintained upon elimination of SPINK1 from stromal cells (Supplementary Fig. 4f), implying SPINK1 drives cancer resistance largely via a caspase-counteracting mechanism. We further applied QVD-OPH and ZVAD-FMK, two potent pan-caspase inhibitors, as well as PAC1 and gambogic acid (GA), two caspase activators, to individually treat PC3 cells before MIT treatment. Cell apoptosis was substantially attenuated when QVD-OPH or ZVAD-FMK was used, even in the presence of SPINK1 (Supplementary Fig. 4g). However, once the procaspase-activating compound PAC1 or GA was present, apoptosis index was markedly increased, offsetting the anti-apoptosis effect of SPINK1. The data were reproduced when docetaxel (DOC), another chemotherapeutic drug that inhibits depolymerization of microtubules, was applied to the system (Supplementary Fig. 4h). In contrast, the overexpression of SPINK1 in stromal cells did not confer survival advantage when cells were exposed to increasing concentrations of genotoxic drugs such as BLEO, suggesting different survival mechanisms between cancer and stromal cells (Supplementary Fig. 4i).

**Fig. 4:**
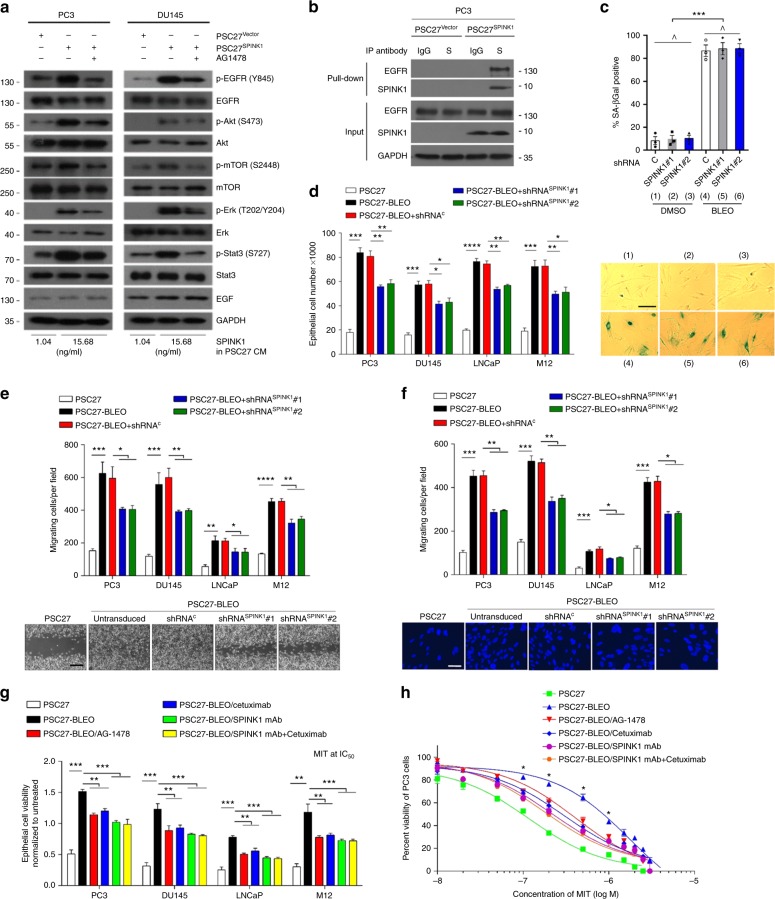
Stromal SPINK1 significantly modifies prostate cancer cell phenotypes in vitro. **a** Immunoblot analysis of EGFR-associated pathways in PCa cells treated by PSC27 CM alone, or with the EGFR inhibitor AG-1478 (2 μM). Total protein per molecule and GAPDH were used as loading control. **b** Immunoprecipitation (IP) followed by immunoblot assay of EGFR and SPINK1 in the whole cell lysates of PC3 treated by the CM of PSC27 sublines for 3 days. S, SPINK1; GAPDH, loading control. **c** Scramble or SPINK1-specific shRNA-transduced PSC27 cells were treated with either DMSO or bleomycin (BLEO) and subject to SA-β-Gal assay. Upper, comparative statistics. Lower, representative images of SA-β-Gal staining. C, scramble. **d** Proliferation measurement of PCa cells treated with the CM of PSC27 sublines for 3 days. **e** Migration assay of PCa cells seeded within transwells in 6-well plates, with cells cultured for 3 days in PSC27 subline CM as indicated in (**c**). Bottom, representative images of PC3 cell migration measured via wound healing assay at 72 h. Scale bars, 100 μm. **f** Invasiveness appraisal of PCa cells across the transwell membrane upon culture with PSC27 subline CM. Bottom, representative images of PC3 cell invasion across the transwell measured at 72 h. Scale bars, 20 μm. **g** Chemoresistance assay of PCa cells cultured with PSC27 subline CM. MIT (mitoxantrone) was applied at the concentration of IC50 value pre-determined per cell line. AG-1478 (2 μM), cetuximab (50 μg/ml), or SPINK1 mAb (1 μg/ml) were applied alongside with PSC27 CM. **h** Dose–response curves (non-linear regression/curve fit) of PC3 cells cultured with PSC27 CM and concurrently treated by a wide range of concentrations MIT. AG-1478 (2 μM), cetuximab (50 μg/ml), and/or SPINK1 mAb (1 μg/ml) were applied with PSC27 CM. Data are mean ± SD and representative of 3 independent experiments, with 3 technical replicates run per cell-based experiment. *P* values were calculated by Student’s *t*-test (**d**–**h**) and one-way ANOVA (**c**) (^*P* > 0.05; *P < 0.05; **P < 0.01; ***P < 0.001)

We next explored the mechanism that permits SPINK1 to confer the pro-survival advantage on cancer cells. As SPINK1 shares ~50% sequence homology with EGF and has 3 intrachain disulfide bridges^32^, we first determined the function of SPINK1 as an EGF-like growth factor. Phosphorylation of EGFR (Y845), Akt (S473), and mTOR (S2448) induced by PSC27^SPINK1^ CM, suggested activation of the PI3K/Akt/mTOR pathway, while phosphorylation of Erk (T202/Y204) and Stat3 (S727) indicated simultaneous activation of MAPK signaling (Fig. 4a). Of note, expression of EGF in cancer cells remained unchanged upon exposure to paracrine SPINK1, precluding the possibility of EGFR activation via an autocrine modality by PCa cells (Fig. 4a). Upon addition of AG-1478, a receptor tyrosine kinase (RTK) inhibitor that specifically targets EGFR^33^, SPINK1-induced EGFR phosphorylation was abrogated, so was activation of both Akt/mTOR and Erk/Stat3 axes (Fig. 4a). Thus, SPINK1-triggered activation of these two signaling pathways was essentially mediated by EGFR, although functional involvement of other RTKs cannot be excluded. SPINK1 elimination by shRNA markedly dampened pathway activation induced by SPINK1^+^ CM, further confirming SPINK1 as a critical paracrine ligand that phosphorylates EGFR and engages multiple intercellular signaling molecules (Supplementary Fig. 4j). Further, immunoprecipitation (IP) showed a strong interaction between SPINK1 and EGFR, as evidenced by the prominent signal in precipitates pulled down by anti-SPINK1 (Fig. 4b).

We subsequently interrogated whether SPINK1, a soluble factor expressed in the entire SASP spectrum of stromal cells, plays a major role in shaping advanced cancer phenotypes. Interestingly, SPINK1 elimination from PSC27 neither delayed nor accelerated cellular senescence (Fig. 4c). Although culturing with the CM of BLEO-treated PSC27 (PSC27-BLEO) increased proliferation, migration, and invasiveness of PCa cells, SPINK1 clearance from stromal cells significantly diminished such malignancy-enhancing capacities of PSC27-BLEO CM, with a reduction of 30–35% in each assayed phenotype (Fig. 4d–f).

SASP factors including WNT16B and SFRP2 have remarkable potential in conferring resistance to cancer cells^12,34^. However, whether SPINK1 plays a similar role in treatment-damaged TME remains unclear. We found cell viability substantially improved upon co-culture with damaged stromal cell-derived CM, although counteracted by ~30% upon SPINK1 elimination or AG-1478 treatment (Supplementary Fig. 4k; Fig. 4g). Upon application of a SPINK1-specific monoclonal antibody (SPINK1 mAb), a significantly decreased cellular viability of PCa cells was observed, with the effect comparable to or even higher than that of either AG-1478 or cetuximab, the latter a food and drug administration (FDA)-approved EGFR-targeting monoclonal antibody (Fig. 4g). Interestingly, co-application of SPINK1 mAb and cetuximab to cell culture achieved an effect that generally resembled that of SPINK1 mAb alone (Fig. 4g), suggesting the addition of cetuximab to SPINK1 mAb did not provide extra benefit. Although PSC27-BLEO CM increased the viability of PC3 exposed to MIT at 0.1–1.0 μM, a range of dose close to its serum concentrations in cancer patients, SPINK1-neutralization offset cancer resistance in a similar way as SPINK1 mAb was co-applied with cetuximab (*P* < 0.01) (Fig. 4h; Supplementary Fig. 4l). Thus, either controlling EGFR or targeting SPINK1 can significantly minimize acquired resistance to chemotherapeutic agents.

### SPINK1 reprograms cancer cell transcriptome-wide expression

**Fig. 5:**
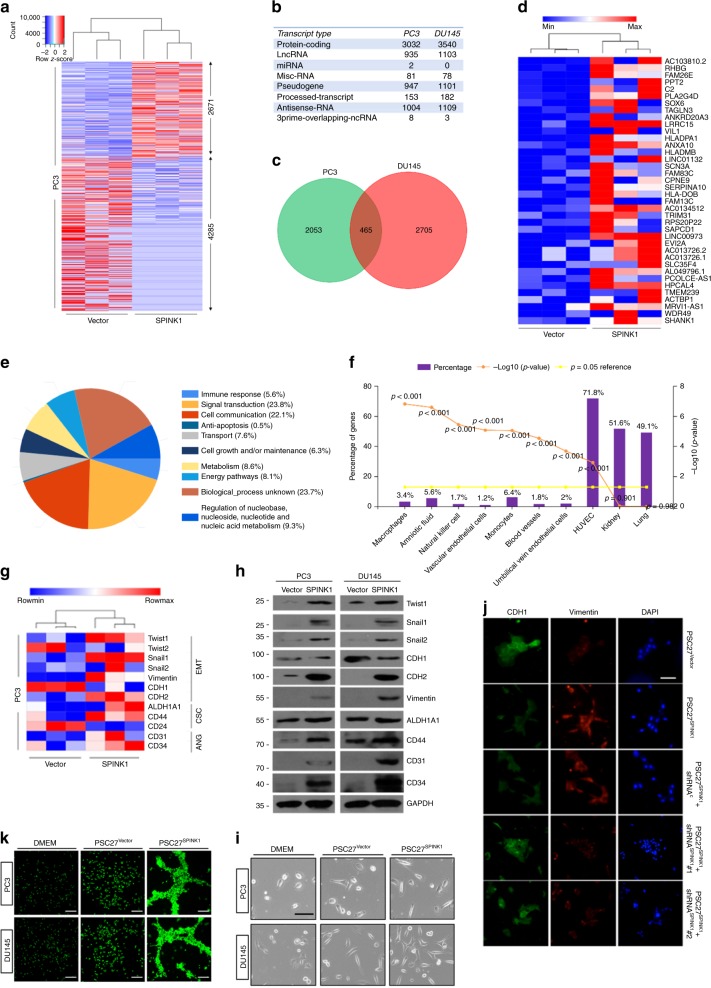
SPINK1 induces profound reprogramming of cancer cell expression and causes phenotypic alteration. **a** Heatmap depicting differentially expressed transcripts in PC3 cells after a 3-day culture with SPINK1^+^ CM from PSC27 cells. Contrasting the control group (vector), 2671 and 4285 genes were upregulated and downregulated, respectively, in cells treated with PSC27^SPINK1^ CM. **b** Statistics of transcripts differentially expressed (fold change either ≥2 or ≤0.5, with *p* < 0.05) in PC3 and DU145 cells upon SPINK stimulation, and classified into typical categories according to functional annotations mapped by Genecode (V27). **c** Venn diagram indicating the overlap of 465 transcripts upregulated in PCa cells upon treatment with SPINK1^+^ CM from PSC27 (2518 and 3170 genes with unique annotations for PC3 and DU145, respectively). **d** Heatmap showing the top 38 upregulated transcripts by both PCa cell lines, sorted according to their expression fold changes in PC3. **e** Pie chart displaying the biological processes associated with 2671 transcripts upregulated by stromal SPINK1 in PC3 cells. **f** Column chart manifesting the expression sites of 2671 transcripts upregulated in PC3 cells after SPINK1 stimulation, with percentage and log10 (*P* value) per specific site indicated on the left and right *Y* axis, respectively. Data derived from by the FunRich program. **g** Heatmap of gene expression signatures associated with phenotypic changes including epithelial–mesenchymal transition (EMT)/cancer stem cell (CSC)/angiogenesis (ANG) after SPINK1 stimulation of PC3 cells. Data were acquired from qRT-PCR assays. **h** Immunoblot assessment of protein level expression of phenotype-associated markers displayed in (**g**). GAPDH, loading control. **i** Representative phase contrast images for morphological changes observed in PC3 and DU145 cells, upon in vitro culture for 3 days with SPINK1^+^ CM from PSC27 cells. Scale bars, 20 μm. **j** Immunofluorescence staining of CDH1 (E-cadherin) and vimentin expressed in PC3 cells treated with PSC27 subline CM. Scale bars, 20 μm. **k** Representative fluorescence images for in vitro tube formation assay to assess angiogenesis of PCa cells placed on the polymerized matrigel. Scale bars, 100 μm. Data of (**g**–**k**) are mean ± SD and representative of 3 independent experiments, with 3 technical replicates performed per cell-based assay

Given the remarkable alterations of cancer cell phenotypes caused by paracrine SPINK1, we next sought to dissect the influence of stromal SPINK1 on cancer cell expression pattern. Data from whole transcriptome RNA sequencing (RNA-Seq) analysis indicated that 6956 transcripts were upregulated or downregulated significantly (2-fold, *P* < 0.05) in PC3 cells by SPINK1 (Fig. 5a), with 8076 transcripts changed in DU145 cells (Supplementary Fig. 5a). Although the vast majority of these transcripts were protein-coding (3032 and 3540 for PC3 and DU145, respectively), there were also molecules that fall into the subcategories of long noncoding RNAs (lncRNAs), microRNA (miRNAs), miscellaneous RNAs (Misc-RNAs), pseudogenes, processed transcripts, antisense RNAs, and 3prime-overlapping-ncRNAs (Fig. 5b).

After mapping the transcripts to a gene ontology (GO) database comprising HPRD, Entrez Gene, and UniProt accession identifiers^35–37^, we noticed there was a portion of 465 co-upregulated transcripts between PC3 (2518) and DU145 (3170) (Fig. 5c). However, upon analysis of the transcripts with a more stringent threshold of 4-fold upregulation with available human genome annotations in PCa cells (627/674 PC3 and 903/940 for PC3 and DU145, respectively), we found an overlap of 38 transcripts which exhibited differential expression fold change and hierarchical order in these two lines (Fig. 5d; Supplementary Fig. 5b–c). Notably, the biological processes of both PC3 and DU145 were remarkably modified, showing a distinct pattern led by alterations in immune response, signal transduction, cell communication, transport, cell growth and/or maintenance, cellular metabolism, energy pathways, and nucleic acid turnaround, although many transcripts remains hitherto undefined (Fig. 5e; Supplementary Fig. 5d). We also observed slightly but significantly enhanced activities associated with anti-apoptosis, in both PC3 and DU145. Further pathway enrichment analysis showed substantial changes in both cellular component and molecular function (Supplementary Fig. 5e–f). We observed prominent expression of genes linked to human endothelial cells (represented by the lineage of HUVEC) when mapping the site of expression of PC3 and DU145 transcripts with a fold change ≥2 (*P* < 0.05) (Fig. 5f; Supplementary Fig. 5g). Thus, SPINK1 induced a striking epithelial-to-endothelial transition (hereby termed EET), suggesting a salient capacity of paracrine SPINK1 in reprogramming the transcriptome of recipient cancer cells.

Further analysis indicated the emergence of advanced malignancies including epithelial–mesenchymal transition (EMT) and cancer stem cell (CSC) appearance, both occurring in parallel with the EET process, as supported by the upregulation of CD31 and CD34, vascular lineage markers indicative of neoangiogenesis^38^ (Fig. 5g; Supplementary Fig. 5h). We noticed enhanced expression of several EMT mediators including the Twist and Snail family of transcription factors (Fig. 5h; Supplementary Fig. 5i). Furthermore, PSC27^SPINK1^ CM-treated cells displayed an apparent change from a typical epithelial to spindle-like or mesenchymal shape, a process accompanied by decreased CDH1 (E-cadherin) but increased CDH2 (N-cadherin) and vimentin expression, implying a prominent EMT transition that occurs in cancer cells upon SPINK1-exposure (Fig. 5h, i; Supplementary Fig. 5i). However, such a tendency was essentially reversed upon SPINK1 depletion from stromal cells, suggesting a pivotal role of SPINK1 in driving such an EMT phenotype (Supplementary Fig. 5i). Immunofluorescence (IF) staining of E-cadherin and vimentin in PCa cells essentially confirmed the potency of SPINK1 in causing an EMT in recipient cancer cells (Fig. 5j).

Besides the EMT-associated alterations, there appeared to be a prominent angiogenesis process induced by stromal SPINK1. In line with the expression of vascularization-related markers (Fig. 5g, h), we noticed the emergence of robust tubular-like structures when cancer cells were exposed to SPINK^+^ CM on a calcein-incorporated basement membrane matrix, suggesting that SPINK1 significantly enhanced cancer cell capacity in forming a capillary tube network (Fig. 5k; Supplementary Fig. 5j–k).

### Targeting SPINK1 sensitizes chemotherapy in vivo

Given the effects of SPINK1 on cancer cell properties in vitro, we next interrogated whether SPINK1 generates any pathological consequences in vivo. To this end, we built tissue recombinants by admixing PSC27 sublines with PC3 cells at a pre-optimized ratio of 1:4 before subcutaneously implanting them to the hind flank of experimental mice with severe combined immunodeficiency (SCID). Animals were measured for tumour size at the end of an 8-week period. Compared with tumours comprising PC3 and PSC27^Vector^, xenografts composed of PC3 and PSC27^SPINK1^ exhibited significantly enhanced volume (Supplementary Fig. 6a). Conversely, knockdown of SPINK1 from PSC27^SPINK1^ cells prior to tumour implantation resulted in considerably reduced tumour volumes.

**Fig. 6:**
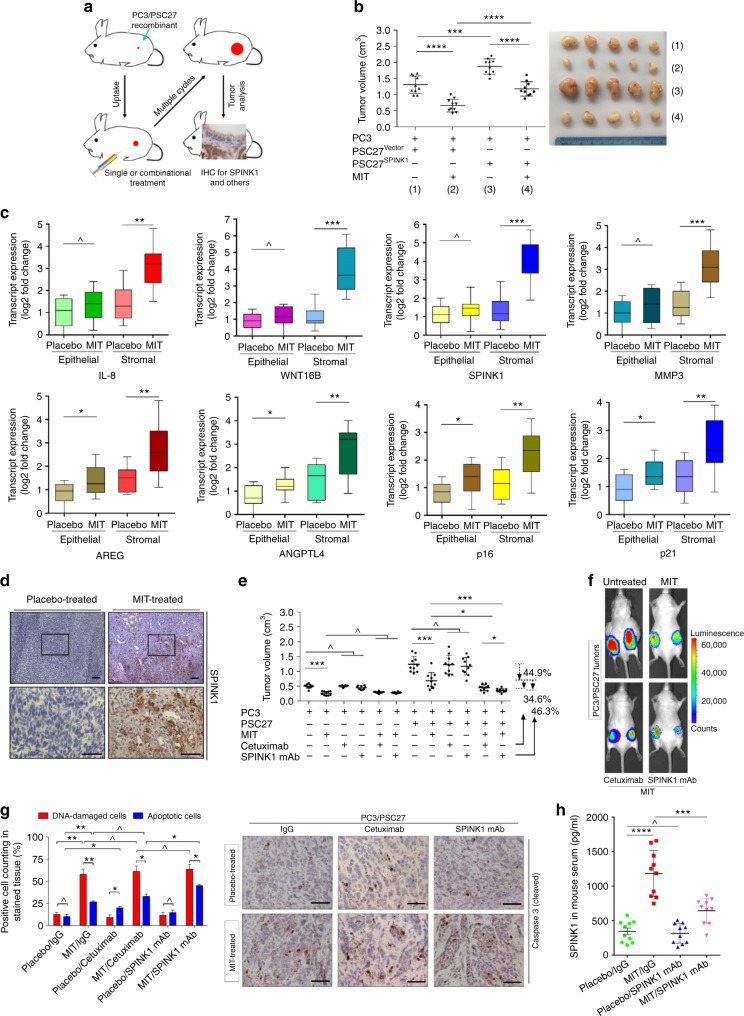
Therapeutically targeting SPINK1 promotes tumour responses to anticancer agents. **a** Experimental workflow for severe combined immunodeficient (SCID) mice. Two weeks after subcutaneous implantation and in vivo uptake of tissue recombinants, animals received single or combinational agents administered as metronomic treatments composed of several cycles. **b** Statistics of tumour end volumes. PC3 cells were xenografted alone or together with PSC27 subline cells to the hind flank of SCID mice. MIT was administered to induce tumour regression. **c** Transcript assessment of several canonical SASP factors expressed in stromal cells isolated from the tumours of SCID mice. Tissues from animals implanted with both stromal and cancer cells were subject to LCM isolation and subsequent assays. **d** Representative IHC images of SPINK1 expression in tissues isolated from placebo or MIT-treated animals. Square regions in the upper images were zoomed into lower images. Scale bars, 100 μm. **e** Statistical comparison of tumour end volumes in animals that underwent several different treatment modalities. Tumour volumes were measured at the end of an 8-week preclinical regimen. **f** Representative bioluminescence images (BLI) of PC3/PSC27 tumour-bearing animals in the preclinical trial. Digital signals were proportional to in vivo luciferase activities measured by an IVIS device. **g** Statistical assessment of DNA-damaged and apoptotic cells in the biospecimens analyzed in (**e**). Values are presented as a percentage of cells positively stained by IHC with antibodies against γ-H2AX or caspase 3 (cleaved). Right, representative IHC images of caspase 3 (cleaved). Biopsies of placebo-treated animals served as negative controls for MIT-treated mice. Scale bars, 100 μm. **h** SPINK1 concentration assessment in circulating blood of experimental mice treated by chemotherapy and/or SPINK1 mAb. Data were derived from human SPINK1-specific ELISA assays. Data are mean ± SD and representative of 3 independent experiments. *P* values were calculated by Student’s *t*-test (**b**, **c**, **e**, **g**, **h**) (^*P* > 0.05; *P < 0.05; **P < 0.01; ***P < 0.001; ****P < 0.0001)

To closely simulate the clinical conditions involving chemotherapeutic agents, we designed a preclinical regimen that incorporates genotoxic therapeutics and/or SPINK1/EGFR inhibitors (Fig. 6a; Supplementary Fig. 6b). Two weeks after cell implantation when stable uptake of tumours by animals was observed, a single dose of MIT or placebo was provided to animals on the first day of 3rd, 5th, and 7th week until the completion of the 8-week regimen. In contrast to placebo, MIT administration generated remarkably decreased tumour sizes regardless of the presence of SPINK1 in stromal cells, validating the efficacy of MIT as a cytotoxic agent (Fig. 6b). However, we noticed significantly enhanced expression of SASP-associated soluble factors including IL-6, IL-8, WNT16B, SPINK1, SFRP2, and MMPs, accompanied by the appearance of senescence markers such as p16 and SA-β-Gal in xenograft tissues comprising PC3/PSC27^Vector^ cells, suggesting the development of an in vivo cellular senescence and typical SASP triggered by MIT (Fig. 6c; Supplementary Fig. 6c–d). IF assays suggested that the stromal cells in xenografts grown subcutaneously were exclusively from implanted human PSC27 cells, not mouse stromal cells migrating to the tumour sites (Supplementary Fig. 6e)

Interestingly, some of the SASP factors such as AREG and ANGPTL4, together with the typical senescence markers including p16 and p21, were co-expressed in stromal and cancer epithelial cells, suggesting drug treatment induced comprehensive in vivo cellular senescence, although the SASP profile seemed to develop differently between stromal and cancer cells (Fig. 6c). Specifically, SPINK1 was apparently induced in the MIT-treated xenografts, with signals predominantly arising from stromal cells (Fig. 6d).

Next, we asked whether therapeutically eliminating SPINK1 from the full SASP spectrum of damaged stoma would further enhance the therapeutic response of tumours. Either cetuximab or SPINK1 mAb was administered alongside MIT since the first dose of preclinical administration. Though MIT treatment caused prominent shrinkage of PC3-only tumours (40.5%), administration of therapeutic antibodies did not show any effect (*P* > 0.05) (Fig. 6e). Interestingly, the antibodies did not provide further benefits even when they were used together with MIT, implying that PC3 tumours grow in a largely EGF/EGFR axis-independent manner in the absence of surrounding stromal cells. Upon implantation of PC3 cells together with their stromal counterparts, tumour volumes significantly increased, consolidating the tumour-promoting effect of stromal cells in vivo (Fig. 6e). However, when animals carrying PC3/PSC27 tumours were treated with MIT, tumour volumes decreased significantly (44.9%, *P* < 0.001). Of note, when either cetuximab or SPINK1 mAb was co-administered with MIT as dual agents, tumour showed further reduction (34.6% and 46.3%, respectively) (Fig. 6e). Bioluminescence imaging (BLI) of xenografts generated with cancer cells stably expressing luciferase (PC3-luc) and stromal cells excluded the potential metastasis of cancer cells from the primary sites, with signals essentially supporting tumour growth patterns in PC3/PSC27 animals (Fig. 6f). The data suggest that conventional chemotherapy combined with stromal component-targeting therapeutics can induce tumour responses more dramatically than chemotherapy alone, and the efficacy of a SPINK1-specific monoclonal antibody is even superior to cetuximab, an anti-EGFR antibody widely used to restrain EGFR^+^ neoplastic cell expansion by promoting their apoptosis in multiple cancer types^39^.

To investigate the mechanism directly responsible for SPINK1-induced cancer resistance, we dissected tumours from animals treated by different agents 7 days after treatment, a time point prior to the development of resistant colonies. Contrasting to placebo, MIT administration caused dramatically increased DNA damage and apoptosis. Although cetuximab alone did not cause DDR, PC3 tumours displayed enhanced cell death, presumably due to the competent binding affinity of cetuximab to EGFR, a property that minimizes cancer cell survival (Fig. 6g). However, when combined with MIT, cetuximab did not exhibit prominent efficacy in enhancing cell apoptosis, implying a reduced cytotoxicity when administered with MIT in these animals. In contrast to cetuximab, however, SPINK1 mAb generated significantly, albeit slightly more apoptotic cells in tumours. IHC staining revealed substantially enhanced caspase 3 cleavage, a typical cell apoptosis hallmark, when SPINK1 mAb was administered (Fig. 6g). Of note, data from ELISA assays on SPINK1 levels in mouse serum indicated that MIT-mediated chemotherapy enhanced circulating SPINK1 amount in vivo, which was substantially reduced when SPINK1 mAb was administered (Fig. 6h).

To further validate these data, we used LNCaP, a second PCa cell line which expresses androgen receptor (AR) and is routinely used as a hormone-dependent cell model. To generate an AR-naive condition, we did not perform castration treatment, but followed the same procedure designed for PC3-tumour animals. LNCaP/PSC27 tumour volumes were markedly reduced when mice were treated by chemotherapy combined with antibodies (Supplementary Fig. 6f). Hence, our results evidently suggest that specific elimination of SPINK1 from the whole spectrum of SASP in the treatment-damaged TME increases tumour response to chemotherapy, a result independent of androgen response or AR signaling of prostate tumours per se.

Given the pronounced efficacy of combinational treatment in preventing prostate tumour progression and minimizing the TME-driven chemoresistance, we expanded the study to a breast tumour by generating xenografts comprising MDA-MB-231 (malignant) and HBF1203 (stromal) cells. Again, MDA-MB-231/HBF1203 tumours reproduced the results observed in PCa preclinical experiments, with combination treatment achieving maximal tumour regression (Supplementary Fig. 6g). Our data suggest that the resistance-antagonizing effects of SPINK1-targeting strategy are not restricted to a specific cancer type, but could be applicable in a pan-cancer manner.

To validate the safety and feasibility of the therapeutic regimens, we performed pathophysiological appraisal which suggested either single or combinational treatment was well tolerated as evidenced by body weight maintenance throughout the therapeutic period (Supplementary Fig. 7a). No significant perturbations in serum levels of creatinine, urea, metabolic activities of liver enzymes were observed (Supplementary Fig. 7b). Data from mice harboring breast tumours treated by DOX/antibody agent(s) and MIT/antibody-treated immunocompetent animals (C57BL/6 background) which displayed no routine blood count changes further supported the findings (Supplementary Fig. 7c–g). Together, these results suggest that combining a SPINK1-targeting agent with conventional chemotherapy has the competence to enhance tumour response without causing severe cytotoxicity.

### SPINK1 is a novel biomarker of the SASP in cancer patients

**Fig. 7:**
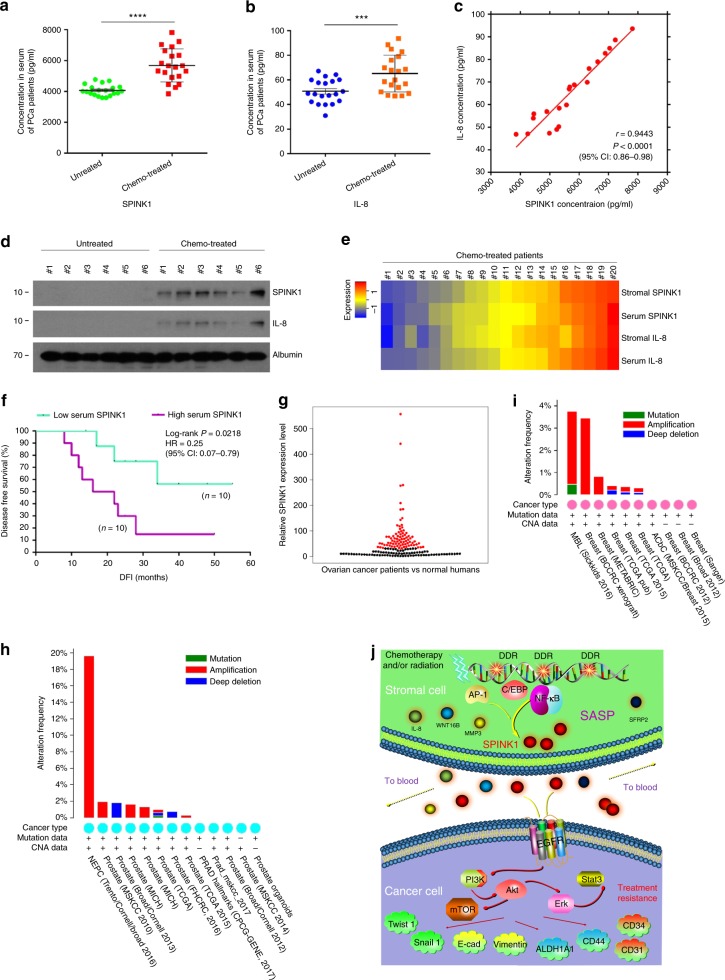
SPINK1 is a novel circulating biomarker indicative of SASP development and predicts the adverse therapeutic outcome of cancer patients. **a** ELISA measurement of SPINK1 protein abundance in the serum of untreated and chemo-treated PCa patients. *n* = 20 per group. **b** ELISA assays of IL-8 protein abundance in patient serum analyzed in (**a**). *n* = 20 per group. **c** Scatterplot showing a correlation between SPINK1 and IL-8 in the serum of individual patients studied in (**a**) and (**b**). Pearson’s correlation coefficient, *P* value and confidence interval indicated. **d** Immunoblot of SPINK1 and IL-8 circulating in the serum of randomly selected PCa patients. Albumin, sample loading control for serum protein. *n* = 6 per group. **e** Heatmap depicting the overall correlation between stromal SPINK1, serum SPINK1, stromal IL-8, and serum IL-8 in chemo-treated patients (*n* = 10). The raw stromal scores derived from independent pathological reading of primary tumour tissues, while serum scores from ELISA assays. **f** Kaplan–Meier survival analysis of chemo-treated PCa patients. Disease-free survival (DFS) stratified according to SPINK1 expression in tumour stroma. DFS represents the length (months) of period calculated from the date of chemotherapy completion to the point of first time disease relapse. **g** TCGA data (Beeswarm graph) show relative expression of SPINK1 in a cohort of ovarian cancer patients (red dots) in contrast to a normal women population (black dots). **h** TCGA data (bar graph) show alterations of SPINK1 in human PCa patients at the genomic level, including mutation, amplification, and deep deletion. Alteration frequency is displayed in percentage. **i** TCGA data (bar graph) display changes of SPINK1 in human BCa patients at the genomic level with the same analysis parameters used in (**h**). **j** Illustrative diagram of SPINK1 expression in the treatment-damaged TME, pathological impact of paracrine SPINK1 on intercellular signaling network of cancer cells and its potential as a therapeutic target and novel biomarker for clinical oncology. Data of (**a**–**e**) are representative of 3 independent experiments. *P* values were calculated by Student’s *t*-test (**a**, **b**), Pearson analysis (**c**), and log-rank (Mantel–Cox) test (**f**) (***P < 0.001; ****P < 0.0001). HR hazard ratio

We next sought to assess whether SPINK1 is experimentally detectable in the circulating system of cancer patients post-chemotherapy. To this end, we collected peripheral blood from PCa patients, including one cohort that experienced chemotherapy and the other that did not. Antigen-specific ELISA analysis of the serum from chemo-treated patients revealed SPINK1 level in the treated cohort significantly higher than that of the treatment-naive group (Fig. 7a). The tendency was phenocopied by a remarkable increase of IL-8, one of the canonical hallmark factors of cellular SASP in these patients (Fig. 7b). Our data suggest the development of an in vivo SASP, the index of which can be measured by quantifying the concurrently expressed soluble factors including but not limited to SPINK1 and IL-8 in the peripheral flood of post-treatment cancer patients.

We then interrogated whether the amount of SPINK1 circulating in the serum is correlated with that of other typical SASP factors such as IL-8 in a same individual patient after cancer treatment. ELISA assay revealed a significantly positive correlation between SPINK1 and IL-8 (Fig. 7c). Immunoblots not only confirmed enhanced SPINK1 and IL-8 levels in the serum of chemo-treated patients, but disclosed their simultaneous changes in vivo, thus validating an intimate correlation between these two factors in the serum of the same individual (Fig. 7d). Additional datasets were obtained from patient cohorts of BCa and CRC, two alternative solid tumour types, thus providing an extra set of evidence (Supplementary Fig. 8a–f).

Next, we expanded the study by longitudinal investigation of these factors in the primary tumour tissue and peripheral blood (20 patients). Strikingly, the cross-organ analysis indicated a remarkable association between the tissue expression and serum level per factor, with the amounts of SPINK1 and IL-8 seemingly in parallel in either primary tissue or circulating blood of each individual patient (Fig. 7e). To establish the appropriateness and reliability of employing SPINK1/IL-8 for in vivo SASP measurement, we selectively acquired stromal cells from the primary tissues of PCa patients via LCM, and analyzed the expression of a subset of signature SASP factors, including MMP1, CXCL3, IL-1β, WNT16B, IL-6, and GM-CSF (Supplementary Fig. 8g). The expression of the vast majority of these factors in stromal cells consistently paralleled the alteration of both SPINK1 and IL-8 in the same tissue. Not surprisingly, however, non-SASP factors such as IL-2/3/5/12 failed to show essential correlation with SPINK1 and IL-8 (Supplementary Fig. 8g). Thus, our data implies that SPINK1 represents one of the major TME-derived soluble factors precisely reflecting the development of an in vivo SASP and can be exploited to assess the SASP magnitude in cancer patients. We observed a negative correlation between SPINK1 plasma level and patient survival in the post-treatment period, further substantiating the pathological impact of SPINK1 as a TME-derived SASP factor, which predicts adverse clinical outcome once the TME experiences irreparable damage by classic interventions (Fig. 7f). As SPINK1 is subject to frequent overexpression, mutation, amplification, and deep deletion in cancer patients as revealed by the TCGA genomics data (Fig. 7g–i), SPINK1 is considered as a potential predictor of disease progression in treatment-naive patients^40,41^. In contrast, routine surveillance of SPINK1 in post-treatment individuals through a noninvasive route such as liquid biopsy provides a novel, handy, and precise strategy for both prognosis and prevention of advanced pathologies in future cancer medicine.

## Discussion

In contrast to many studies that focus on cell-autonomous or intrinsic mechanisms of cancer resistance, we highlight the functional significance of the treatment-damaged TME in conferring acquired resistance to therapeutic agents, whereby SPINK1 plays a major role in mediating the tumour–stroma communication. Mounting evidence proved that stromal expression of soluble factors including HGF, WNT16B, and TNF-α in the TME has a significant correlation with cancer resistance to chemotherapy, radiation, and targeted agents^3,5,12,42^. Our study substantiates the pathological influence of the TME on disease exacerbation by producing soluble factors such as SPINK1.

In the local microenvironment, repairable DNA damage does not result in comprehensive cytokine secretion, but more extensive genotoxic stress causes a persistent DDR and initiates the SASP, a hallmark of cellular senescence that develops after exposure of cells to inherent or environmental insults including anticancer therapeutics^7,43^. The SASP is frequently activated by DNA damage, modulated by stress-response kinases including CHK2, ATM, and NBS1, and consolidated by a handful of SASP factors such as the inflammatory cytokines IL-6 and IL-8^43–46^. While multiple data suggest that intracellular molecules including p38, mTOR, and GATA4 regulate the SASP development, they eventually cause NF-kB activation^47–49^. Here, we show that DNA damage-induced expression of SPINK1 in human stromal cells involves not only NF-kB, but also C/EBP family and AP-1. Although former studies suggested the presence of C/EBP binding sites in the promoters of SASP factors, particularly a subset of CXCR2 ligands including IL-6, IL-8, GROα/β/γ (CXCL1/2/3), ENA-78 (CXCL5), and NAP2 (CXCL7)^46^, we extended the range of C/EBP-regulated SASP factors by adding SPINK1. Interestingly, a recent study reported that c-Myb and C/EBP co-regulate osteopontin (OPN) and many other SASP factors^50^, further suggesting the regulatory complexity.

SPINK1^+^ PCa cell-derived SPINK1 enhances cell proliferation and invasion via autocrine and paracrine signaling in vitro, while promoting cell intravasation across chick chorioallantoic membrane and tumour growth in vivo^29^. SPINK1 binds to EGFR, whereas inhibiting SPINK1 attenuates the activation of EGFR downstream molecules including MEK, Erk, and Akt^29,51^. A recent study indicated that SPINK1 promotes the proliferation of BRL-3A, a rat liver hepatocyte cell line, via activation of EGFR and its downstream p38, Erk, and JNK pathways^52^. Of note, SPINK1 and EGFR are significantly co-overexpressed in a cohort of PCa patients, whereby SPINK1 induces EMT by engaging the MAPK/Mek/Erk pathway^53^. In this study, we found stromal cell-derived SPINK1 not only activates Akt/mTOR, Mek/Erk/Stat3 pathways, signaling branches downstream of EGFR, but also generates profound impact on cancer cell transcriptomics. First, our data suggested the emergence of EMT, a phenotypic switch evidenced by concurrent expression changes of EMT-specific markers. Second, we observed gene expression pattern indicative of the development of CSC. Third, an important step of cancer evolution, neoangiogenesis, emerged upon the treatment of cancer cells with stromal SPINK1. Of note, HUVEC arised saliently as the site where the majority of SPINK1-activated genes were intensively expressed, a unique feature rarely reported for SASP factors. Our data suggest the phenotypic transition from cancer epithelial cells more specifically toward endothelial cells (EET), as supported by both bioinformatics prediction and protein expression analysis. Altogether, the expression of key molecules associated with tumour-promoting pathways suggests enhanced malignancy driven by paracrine SPINK1 and indicates an adverse prognosis in the post-therapy stage.

SPINK1 expression at physiological level is confined to gastrointestinal (GI) tissues, with its overproduction mostly preserved in GI malignancies^54,55^. A former study using cancer outlier profile analysis (COPA) reported that ~50% of PCa patients of PSA-screened Caucasian cohorts harbor genetic rearrangements between the 5′ untranslated region of TMPRSS2, an androgen responsive gene, and the ETS (E26 transformation-specific) family of transcription factors including ERG, ETV1, and ETV4^56^. Although aberrant expression of SPINK1 was identified as an outlier factor and indicative of a subset of ETS-negative patients, the ratio is limited to approximately 10% of total PCa population^20^. However, a transcriptional circuit consisting of hepatocyte nuclear factor 4-gamma (HNF4G) and hepatocyte nuclear factor 1-alpha (HNF1A) was recently found to regulate a PCa-GI transcriptome involving enhanced SPINK1 expression, a case linked with AR therapy resistance^55^. Here, we found SPINK1 is significantly induced by genotoxic treatments such as genotoxic chemotherapy, with the expression mostly arising in the damaged stroma. Contrasting the limited therapeutic effect of SPINK1-targeting agents projected for SPINK1^+^ individuals in PCa patients, SPINK1 mAb-based therapies against the TME take advantage of circumventing the need to stratify cancer patients according to any molecularly defined attributes. Intriguingly, SPINK1 mAb manifested superior efficacy when compared with the conventional EGFR mAb cetuximab, regardless in vitro or in vivo. It remains elusive why a SPINK1-specific mAb can be more effective than an EGFR-oriented mAb in reducing cancer malignancy. However, it was reported that certain EGFR monoclonal antibodies such as mAb 806 only binds a transitional form of EGFR after the cell surface receptor untethers but before forming an active oligomer^57^. AG-1478 increases mAb 806 binding to EGFR and induces the formation of inactive EGFR dimers that can sequester its ligand, while the interaction between AG-1478 and mAb 806 generates additive effect in tumour treatment^57^. Thus, SPINK1 mAb holds the advantage by directly neutralizing SPINK1 protein in the extracellular space, as the ligand does not need to undergo conformational change to allow maximal intermolecular interplay between the antibody and the antigen as exemplified by the case of EGFR. The elimination of SPINK1 from stromal cells generated significant effect by reducing cancer cell malignancy, both substantiating SPINK1 as one of the major factors in the SASP spectrum in shaping cancer plasticity, and suggesting the exploitable value of targeting SPINK1 to minimize cancer resistance acquired from the TME. On the other hand, data from in vitro assays indicated that SPINK1 effects are mediated predominantly through EGFR, but the involvement of other RTKs yet remains possible, an issue raised by former studies^29,58^ which can alternatively explain the higher efficacy of SPINK1 mAb in antagonizing cancer progression than an EGFR-targeting antibody.

It is not surprising that multiple SASP factors released from the damaged stroma can contribute to cancer resistance in therapeutic settings, including but not limited to WNT16B and SFRP2^12,34^. Here, we explored the possibility of minimizing cancer resistance by targeting the major SASP factors which act as the most critical players in driving acquired resistance, with SPINK1 emerging as one of such targetable molecules. Our results not only provide a rationale for the development of humanized mAbs to SPINK1, but imply the technical feasibility of restraining cancer resistance by delivering a panel of humanized mAbs against the key SASP factors, to significantly improve therapeutic outcome in cancer clinics.

We discovered SPINK1 in circulating blood of both experimental animals and human patients post-chemotherapy. In this aspect, SPINK1 can serve as a potential biomarker indicative of a treatment-damaged TME. However, one caveat to our study was the animal model used for the collection of in vivo data. Although male mice were consistently chosen for tumour implantation at their hind flank, we have to admit that the microenvironments of human prostate, human breast, murine prostate, and murine mammary are all different. Despite the complexity and variability of the microenvironment across organ and/or species types, we speculate that SPINK1 expression is not limited to the TME of a specific cancer type such as PCa or BCa, but could be universal across multiple malignancies, a phenomenon that should be paid substantial attention. Although well-validated assays of soluble factors are developed, comprehensive subtyping using SPINK1-based assays for pan-cancer investigation remains an unexplored but exciting area in translational medicine.

Together, our study unmasks SPINK1 as a key SASP factor in the treatment-damaged TME that confers acquired resistance throughout therapeutic regimens (Fig. 7j). As the response of the TME to anticancer agents can be molecularly identified by monitoring the circulating SASP factors in patient blood, SPINK1 represents both a biomarker that merits further investigation to establish its potential as a standard of cancer surveillance and a target to develop humanized antibodies for clinical trials. In the future, timely and precise assessment of the host TME with multiplexed and multimodality biomarkers holds the potential to profile molecular sub-classification, which helps predict the patient response to conventional, customized, or personalized therapies.

## Methods

### Cell culture

Primary normal human prostate stromal cell line PSC27 and breast stromal cell line HBF1203 were maintained in stromal complete medium as described^12^. Prostate epithelial cell lines PC3, DU145, LNCaP and breast epithelial cell lines MCF7, MDA-MB-231, MDA-MB-468, T47D, and BT-474 (ATCC) were routine cultured with RPMI 1640 (10% FBS). BPH1 was isolated from prostatic tissue with benign hyperplasia and immortalized by SV40-LT^59^, while M12 was derived from BPH1 but phenotypically neoplastic and metastatic^60^. All cell lines were routinely tested for mycoplasma contamination and authenticated with STR assays.

### Vectors, viruses, and infection

Full length human SPINK1 sequence was cloned into pLenti-CMV/To-Puro-DEST2 as described^12^. Small hairpin RNAs (shRNA) targeting SPINK1 (1#, sense strand 5′-GAAGAGAGGCCAAATGTTATTCAAGAGATAACATTTGGCCTCTCTTCTTTTTG-3′, anti-sense strand 5′-AAAAAGAAGAGAGGCCAAATGTTATCTCTTGAA TAACATTTGGCCTCTCTTCA-3′; 2#, sense strand 5′-CCAAGATATATGACCCTGTTTCAAGAGAACAGGGTCATATATCTTGGTTTTTG-3′, anti-sense strand 5′-AAAAACCAAGATATATGACCCTGTTCTCTTGAA ACAGGGTCATATATCTTGGA-3′; scramble, sense strand 5′-TAGCGACTAAACACATCAATTCAAGAGATTGATGTGTTTAGTCGCTATTTTTTG-3′, anti-sense strand 5′-AAAAAATAGCGACTAAACACATCAATCTCTTGAATTGATGTGTTTAGTCGCTAA-3′) were cloned in pLKO.1-Puro vector (Addgene). Upon production by 293T cells, lentiviral titers were adjusted to infect ~90% of cells. Stromal cells were infected overnight in the presence of polybrene (8 μg/ml), allowed to recover for 48 h and selected for 72 h before used for further analysis. For expression of target genes in either stromal or epithelial cells, total RNA was prepared and subject to qRT-PCR assays (primers listed in Supplementary Table 4), immunoblot, and immunofluorescence analysis.

### Cell treatments

Stromal cells were grown until 80% confluent (CTRL) and treated with 50 μg/ml bleomycin (BLEO), 500 nM mitoxantrone (MIT), 10 μM satraplatin (SAT), 10 μM doxorubicin (DOX), 100 μM cisplatin (CIS), 200 μM carboplatin (CARB), 0.6 mM hydrogen peroxide (HP), or γ-radiation by a ^137^Cs source at 743 rad/min for 10 Gy (RAD). After treatment, the cells were rinsed briefly with PBS and allowed to stay for 7–10 days prior to performance of various examinations.

### Human cancer patient recruitment and biospecimen analysis

The administration of chemotherapeutic agents was performed for primary PCa patients (Clinical trial no. NCT03258320), infiltrating ductal BCa patients (NCT02897700), and primary CRC patients (NCT00643877), by following the CONSORT 2010 Statement (updated guidelines for reporting parallel group randomized trials). Patients with a clinical stage ≥I subtype A (IA) (T1a, N0, M0) of primary cancer but without manifest distant metastasis were enrolled into the multicentred, randomized, double-blinded, and controlled pilot studies. Age between 40 and 75 years with histologically proven PCa, age ≥18 years with histologically proven infiltrating ductal BCa, or age <75 years with histologically proven CRC was required for recruitment into the clinical cohorts. Data regarding tumour size, histologic type, tumour penetration, lymph node metastasis, and TNM stage were obtained from the pathologic records. Tumours were processed as FFPE biospecimens and sectioned for histological assessment, with alternatively prepared OCT-frozen chunks processed via LCM for gene expression analysis. Specifically, stromal compartments associated with glands and adjacent to cancer epithelium were separately isolated from tumour biopsies before and after chemotherapy using an Arcturus (Veritas Microdissection) laser capture microscope following previously defined criteria^12^. The immunoreactive scoring (IRS) gives a range of 1–4 qualitative scores according to staining intensity per tissue sample. Categories for the IRS include 0–1 (negative), 1–2 (weak), 2–3 (moderate), 3–4 (strong)^61^. The diagnosis of PCa, BCa, and CRC tissues was confirmed based on histological evaluation by independent pathologists. Randomized control trial (RCT) protocols and all experimental procedures were approved by the Institutional Review Board of Shanghai Jiao Tong University School of Medicine, with methods carried out in accordance with the official guidelines. Informed consent was obtained from all subjects and the experiments conformed to the principles set out in the WMA Declaration of Helsinki and the Department of Health and Human Services Belmont Report.

### Histology and immunohistochemistry

Formalin-fixed paraffin-embedded (FFPE) tissue sections of 7 μm were deparaffinized in xylenes and rehydrated through a graded series of alcohols. Routine histology appraisal was performed with hematoxylin and eosin staining. For immunohistochemical (IHC) evaluation, FFPE sections experienced antigen retrieval with sodium citrate, incubation with 3% H_2_O_2_, treatment with avidin/biotin blocking buffer (Vector Laboratories), and then 3% BSA for 30 min. Staining with primary and secondary antibodies was conducted at 4 °C for overnight and at room temperature for 60 min, respectively. Sections were incubated with a H_2_O_2_-diaminobenzidine (DAB) substrate kit (Vector, SK-4100). Samples were counterstained with hematoxylin, dehydrated, and mounted. IHC images were obtained using an upright microscope (Olympus BX51). Brown staining indicated the immunoreactivity of samples.

### RNA-Seq and bioinformatics analysis

Total RNA samples were obtained from PC3 and DU145 cells cultured with CM of either PSC27^Vector^ or PSC27^SPINK1^. Sample quality was validated by Bioanalyzer 2100 (Agilent), and RNA was subjected to sequencing by Illumina HiSeq X 10 with gene expression levels quantified by the software package RSEM (https://deweylab.github.io/RSEM/). Briefly, rRNAs in the RNA samples were eliminated using the RiboMinus Eukaryote kit (Qiagen, Valencia, CA, USA), and strand-specific RNA-seq libraries were constructed using the TruSeq Stranded Total RNA preparation kits (Illumina, San Diego, CA, USA) according to the manufacturer's instructions before deep sequencing.

Pair-end transcriptomic reads were mapped to the reference genome (GRCh38/hg38) with reference annotation from Gencode v27 using the Bowtie tool. Duplicate reads were identified using the Picard tools (1.98) script mark duplicates (https://github.com/broadinstitute/picard) and only non-duplicate reads were retained. Reference splice junctions are provided by a reference transcriptome (Ensembl build 73)^62^. FPKM values were calculated using Cufflinks, with differential gene expression called by the Cuffdiff maximum-likelihood estimate function^63^. Genes of significantly changed expression were defined by a false discovery rate (FDR)-corrected *P* value < 0.05. Only Ensembl genes 73 of status “known” and biotype “coding” were used for downstream analysis.

Reads were trimmed using Trim Galore (v0.3.0) (http://www.bioinformatics.babraham.ac.uk/projects/trim_galore/) and quality assessed using FastQC (v0.10.0) (http://www.bioinformatics.bbsrc.ac.uk/projects/fastqc/). Differentially expressed genes were subsequently analyzed for enrichment of biological themes using the DAVID bioinformatics platform (https://david.ncifcrf.gov/), the Ingenuity Pathways Analysis program (http://www.ingenuity.com/index.html). Raw data of RNA-Seq were deposited in the NCBI Gene Expression Omnibus (GEO) database under the accession code GSE108545.

Venn diagrams: Venn diagrams and associated empirical *P*-values were generated using the USeq (v7.1.2) tool IntersectLists^64^. The *t*-value used was 22,008, as the total number of genes of status “known” and biotype “coding” in Ensembl genes 73. The number of iterations used was 1000.

RNA-Seq heatmaps: For each gene, the FPKM value was calculated based on aligned reads, using Cufflinks^63^. *Z*-scores were generated from FPKMs. Hierarchical clustering was performed using the R package heatmap.2 and the distfun = “pearson” and hclustfun = “average”.

Principal component analysis: Principal component analysis (PCA) was performed using the FPKM values of all Ensembl genes 73 of status “known” and biotype “coding”.

Site of expression: Site of expression of differently expressed genes was assessed with FunRich, a stand-alone software designed for functional enrichment and interaction network analysis of genes and proteins in normal tissues, cancer tissues, cell types, and cell lines^65^.

### SPINK1 promoter characterization and ChIP-PCR assays

A 3953 bp genomic region upstream of human SPINK1 ORF was analyzed for core NF-κB binding sites. The immediate 5′ upstream sequences containing putative NF-κB binding sites were amplified from the human genomic DNA. Four regions that encompass augmenting numbers of NF-κB binding sites were cloned into a luciferase reporter vector pGL4.22 (Promega). A NAT11-Luc2CP-IRES-nEGFP construct was used as a positive control as described^12^. After reporter constructs were co-transfected with a pRL-TK vector for transfection normalization, cells were treated with 40 ng/ml TNF-α or 20 ng/ml IL-1α for 3 h, or 10 μM satraplatin for 1 day. Luciferase activity was measured using the Dual-Luciferase Reporter Assay System (Promega). The NF-κB inhibitor Bay 11-7082 (5 μM), c/EBP antagonist BA (10 μM), c-Fos/AP-1 suppressor T-5224 (10 μM), and AP-1 inhibitor SR11302 (3 μM) were applied, with cells lysed 3 days later for luciferase assays.

For ChIP-PCR assays, four primer sets were designed to amplify short sequences within the approximal promoter region [primer set #1 (−482 to −259) forward 5′-CTACTGAAATCACAGTGAAGTATAG-3′, reverse 5′-CTGTTCATTGCATCCTGCTAT-3′; primer set #2 (−1870 to −1625) forward 5′-GACCAGTCTGGCCAACATGG-3′, reverse 5′-CCTCATGCTGTATGTTAGATATTCAGAC-3′; primer set #3 (−1917 to −1773) forward 5′-TACTTTGGGAGGCCGAGGCAG-3′, reverse 5′-CTCCCGAGTAGCTGGGATTACAGG-3′; primer set #4 (−4000 to −3798) forward 5′-TTTAAGAACCTACTATGTGTTTGG-3′, reverse 5′-GAAACTCTTGGACACTTTGAG-3′]. Additionally, two specific primer sets were employed to amplify regions within the promoters of the human IL-6 (forward 5′-AAATGCCCAACAGAGGTCA-3′, reverse 5′-CACGGCTCTAGGCTCTGAAT-3′) and IL-8 (forward 5′-ACAGTTGAAAACTATAGGAGCTACATT-3′, reverse 5′-TCGCTTCTGGGCAAGTACA-3′) genes, respectively, which encompass known NF-κB binding sites^12^. ChIP assays were then performed on PSC27 cells of early passage (p10) and those treated by 50 μg/ml bleomycin in culture. A sample of formalin-fixed sheared chromatin (DNA fragments at an average size of ~500 bp) from these cells was used as “input DNA” for control amplification. Fixed chromatin was immunoprecipitated using mouse monoclonal anti-p65 antibody (Santa Cruz) and DNAs were extracted from the immunoprecipitates and amplified using the primer sets described above. Control immunoprecipitations were carried out with a mouse IgG, which essentially yielded no reaction products.

### Immunoblot and immunofluorescence analysis

Whole cell lysates were prepared using RIPA lysis buffer supplemented with protease/phosphatase inhibitor cocktail (Biomake). Nitrocellulose membranes were incubated overnight at 4 °C with primary antibodies list in Supplementary Table 5, with HRP-conjugated goat anti-mouse or -rabbit serving as secondary antibodies (Vazyme). For immunofluorescence analysis, cells were fixed with 4% formaldehyde and permeabilized before incubation with primary and secondary antibodies, each for 1 h. Upon counterstaining with DAPI (0.5 μg/ml), samples were examined with an Imager.A1 (Zeiss) upright microscope to analyze specific gene expression. Uncropped scans of the most important blots are provided in Supplementary Fig. 9.

### In vitro cell phenotypic characterization

For proliferation assays of cancer cells, 2 × 10^4^ cells were dispensed into 6-well plates and co-cultured with conditioned medium (CM) from the stromal cells. Three days later, cells were digested and counted with hemacytometer. For migration assays, cells were added to the top chambers of transwells (8 μm pore), while stromal cell CM were given to the bottom. Migrating cells in the bottom chambers were stained by DAPI 12–24 h later, with samples examined with Axio Observer A1 (Zeiss). Invasion assays were performed similarly with migration experiments, except that transwells were coated with basement membrane matrix (phenol red free, Corning). Alternatively, cancer cells were subject to wound healing assays conducted with 6-well plates, with healing patterns graphed with bright field microscope. For chemoresistance assays, cancer cells were incubated with stromal cell CM, with the chemotherapeutic agent MIT provided in wells for 3 days at each cell line’s IC50, a value experimentally predetermined. Commercial SPINK1 (Human) recombinant protein (Abnova, Cat. No. H00006690-P01) was employed at 100 ng/ml as a positive control for cell-based assays.

Tube formation (in vitro angiogenesis) assay was based on the ability of reprogrammed cancer cells to form 3D capillary-like tubular structures on a basement membrane matrix. Briefly, PC3 and DU145 cells (3.5 × 10^4^ cells/200 μl/cm^2^) were suspended with LSGS-supplemented media 200PRF, loaded onto 12-well plates pre-coated with 50 μl of Geltrex^TM^ (Thermo Fisher). Plates were incubated at 37 °C for 8 h and cells were stained with calcein-AM to measure cell viability and analyze tube formation. Cultures were photographed by phase contrast or fluorescence microscopy.

### Co-immunoprecipitation

Cells were rinsed twice with cold PBS, then lysed on ice for 20 min in 1 ml of lysis buffer (40 mM HEPES at pH 7.5, 120 mM NaCl, 1 mM EDTA, 10 mM pyrophosphate, 10 mM glycerophosphate, 50 mM NaF, 0.5 mM orthovanadate, EDTA-free protease inhibitors) containing 0.3% CHAPS. Four micrograms of antibody specific to SPINK1 (Abnova) were added to the cleared cellular lysates and incubated with rotation for overnight. Then, 50 μl of protein A/G-agarose beads (Pierce) were added and the incubation continued for 12 h at 4 °C. Immunoprecipitates captured with the beads were washed thrice with the CHAPS lysis buffer and twice by wash buffer A (50 mM HEPES at pH 7.5, 150 mM NaCl, protease phosphatase inhibitors included), and boiled in 4× SDS sample buffer prior to electrophoresis and immunoblotting.

### Production and purification of recombinant human SPINK1

The human SPINK1 ORF, excluding the signal peptide sequence (69 bp), was subcloned from pLenti-SPINK1-Puro (described as above) downstream of the Met-Arg-Gly-Ser-His_6_ (MRGSH_6_) coding sequence between the BamHI/HindIII restriction sites of the pQE9 expression vector (Qiagen). The new construct pQE9-SPINK1 was sequenced to verify integrity, before the production of a recombinant protein by M15 *Escherichia coli* cells induced with 0.4 mM isopropyl-1-thio-D-galactopyranoside (IPTG) in culture. Bacterial lysates were centrifuged, with the supernatants purified by affinity chromatography using Co^2+^-agarose resin (Qiagen). Bacterially expressed SPINK1 recombinant protein was separated using Sephadex-G50 (Sigma) by size exclusion chromatography. SPINK1 (MW 6.2 kDa) was then concentrated by ultrafiltration using Amicon Ultra NMWL 3 kDa centrifugal filter units (Merck Millipore).

### In vivo SASP assessment of patients and ELISA assays

Sections of clinical biospecimens or animal tissues were processed via LCM for gene expression analysis. Specifically, stromal compartments associated with glands in patient tumour samples were separately isolated using an Arcturus (Veritas Microdissection) laser capture microscope following the criteria defined formerly^12^. For tumours grown from xenografts composed of human cells, OCT sections were first H&E stained to determine the location of stromal cells and the stroma–epithelium border, with cell lineages then separately acquired by LCM. Transcript levels of human SASP canonical factors including IL-6, IL-8, WNT16B, SPINK1, SFRP2, MMP1, MMP3, and MMP12 were measured by qRT-PCR (primers listed in Supplementary Table 5).

Peripheral blood samples from cancer individuals with matched FFPE or frozen tumour samples were collected in EDTA tubes and centrifuged at 2000*g* for 10 min at room temperature within 1 h of clinical acquisition to prepare high-quality serum. SPINK1 and IL-8 proteins in serum of cancer patients were subject to quantification by antigen-specific ELISA kits (R&D Systems, DY7496/DY208) according to manufacturer’s instructions. Detection limits for these factors were 10 pg/ml.

### Experimental animals and preclinical studies

All animals were maintained in a specific pathogen-free (SPF) facility, with NOD/SCID (Charles River and Nanjing Biomedical Research Institute of Nanjing University) mice at an age of approximately 6 weeks (~20 g body weight) used. Ten mice were incorporated in each group, and xenografts were subcutaneously generated at the hind flank upon anesthesia mediated by isoflurane inhalation. Stromal cells (PSC27 or HBF1203) were mixed with cancer cells (PC3, LNCaP, or MDA-MB-231) at a ratio of 1:4 (i.e., 250,000 stromal cells admixed with 1,000,000 cancer cells to make tissue recombinants before implantation in vivo). Animals were sacrificed at 2–8 weeks after tumour xenografting, according to tumour burden or experimental requirements. Tumour growth was monitored weekly, with tumour volume (*v*) measured and calculated according to the tumour length (*l*), width (*w*), and height (*h*) by the formula: *v* = (*π*/6) × ((*l* + *w* + *h*)/3)^3^. Freshly dissected tumours were either snap-frozen or fixed to prepare FFPE samples. Resulting sections were used for IHC staining against specific antigens or subject to hematoxylin/eosin staining.

For chemoresistance studies, animals received subcutaneous implantation of tissue recombinants as described above and were given standard laboratory diets for 2 weeks to allow tumour uptake and growth initiation. Starting from the 3rd week (tumours reaching 4–8 mm in diameter), MIT (0.2 mg/kg doses), DOX (doxorubicin, 1.0 mg/kg doses), therapeutic antibodies (cetuximab or SPINK1 mAb, 10.0 mg/kg doses, 200 μl/dose), or vehicle controls was administered by body injection (chemicals via intraperitoneal route, antibodies through tail vein), on the first day of 3rd, 5th, and 7th week, respectively. Upon completion of the 8-week therapeutic regimen, animals were sacrificed, with tumour volumes recorded and tissues processed for histological evaluation.

At the end of chemotherapy and/or targeting treatment, animals were anaesthetized and peripheral blood was gathered via cardiac puncture. Blood was transferred into a 1.5 ml Eppendorf tube and kept on ice for 45 min, followed by centrifugation at 9000*g* for 10 min at 4 °C. Clear supernatants containing serum were collected and transferred into a sterile 1.5 ml Eppendorf tube. All serum markers were measured using dry-slide technology on IDEXX VetTest 8008 chemistry analyzer (IDEXX). About 50 μl of the serum sample was loaded on the VetTest pipette tip followed by securely fitting it on the pipettor and manufacturer’s instructions were followed for further examination.

All animal experiments were performed in compliance with NIH Guide for the Care and Use of Laboratory Animals (National Academies Press, 2011) and the ARRIVE guidelines, and were approved by the Institutional Animal Care and Use Committee (IACUC) of the University of Washington or Shanghai Institutes for Biological Sciences, Chinese Academy of Sciences.

### Statistics

All in vitro experiments were performed in triplicates, and animal studies were performed with 10 mice per group. Unless otherwise indicated, data in the figures are presented as mean ± SD, and statistical significance was determined by unpaired two-tailed Student’s *t* test. Cox proportional hazards regression model and multivariate Cox proportional hazards model analysis were performed with statistical software SPSS (23). Statistical significance was determined by two-tailed Student’s *t* test, one- or two-way ANOVA, Pearson’s correlation coefficients test, Kruskal–Wallis, log-rank test, Wilcoxon–Mann–Whitney test, or Fisher’s exact test. For all statistical tests, a *P* value < 0.05 was considered significant.

To determine sample size, we began by setting the values of type I error (*α*) and power (1 − *β*) to be statistically adequate: 0.05 and 0.80, respectively^66^. We then determine *n* on the basis of the smallest effect we wish to measure. If the required sample size is too large, we chose to reassess the objectives or to more tightly control the experimental conditions to reduce the variance.

## Electronic supplementary material

Supplementary Information

## Data Availability

The raw RNA-Seq data have been deposited in the Gene Expression Omnibus database (accession code GSE108545). All sequencing experiments were performed as independent triplicates, and the RNA-Seq data referenced during the study are available in a public repository (https://www.ncbi.nlm.nih.gov/geo/). The authors declare that all the other data supporting the findings of this study are available within the article or its Supplementary Information files and from the corresponding author upon reasonable request.
