# Supplementary material for: Targeting SPINK1 in the damaged tumour microenvironment alleviates therapeutic resistance
Source: Nat Commun. 2018 Oct 17;9:4315. doi: 10.1038/s41467-018-06860-4 (PMC6193001; doi:10.1038/s41467-018-06860-4)
Supplement: Supplementary file 1 — Supplementary Information [file 41467_2018_6860_MOESM1_ESM.pdf]

Targeting SPINK1 in the damaged tumour microenvironment alleviates therapeutic resistance

Sun et al.

File Name: Supplementary Information

Description: Supplementary Figures and Supplementary Tables

****

Supplementary Figures: Supplementary Figure 1-8

Supplementary Tables: Supplementary Table 1-5

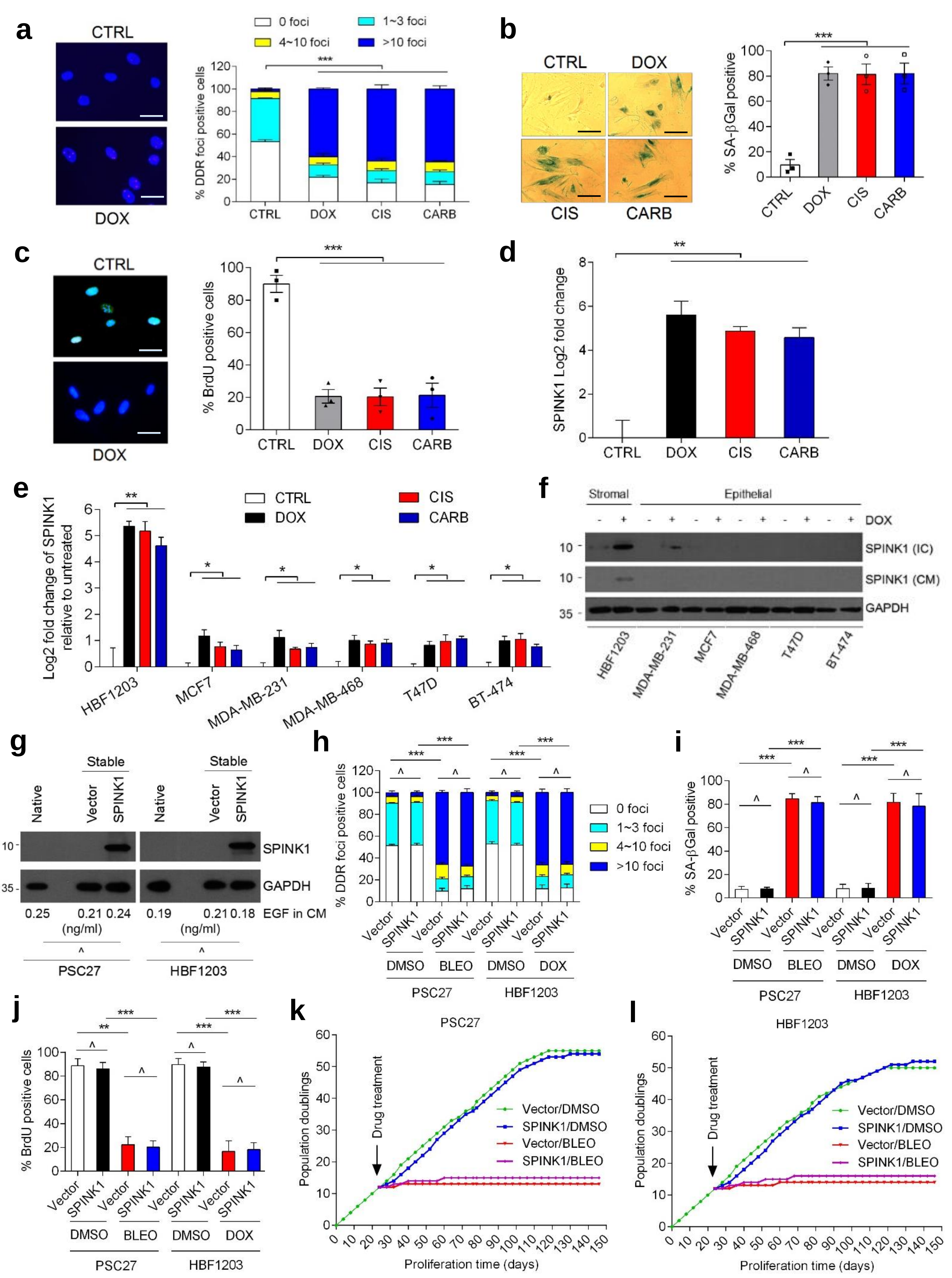
a
CTRL
DOX
0 foci
1~3 foci
4~10 foci
>10 foci
% DDR foci positive cells
CTRL DOX CIS CARB
b
CTRL DOX
CIS CARB
% SA-βGal positive
c
CTRL
DOX
% BrdU positive cells
d
SPINK1 Log2 fold change
e
Log2 fold change of SPINK1 relative to untreated
HBF1203 MCF7 MDA-MB-231 MDA-MB-468 T47D BT-474
f
Stromal
Epithelial
DOX
SPINK1 (IC)
SPINK1 (CM)
GAPDH
g
Stable
Native Vector SPINK1
SPINK1
GAPDH
0.25 0.21 0.24 0.19 0.21 0.18 EGF in CM
(ng/ml)
PSC27
HBF1203
h
i
j
Vector SPINK1
DMSO BLEO DMSO DOX
PSC27 HBF1203
k
l
PSC27
HBF1203
Drug treatment
Population doublings
Proliferation time (days)
Vector/DMSO
SPINK1/DMSO
Vector/BLEO
SPINK1/BLEO

Supplementary Figure 1. SPINK1 expression is upregulated in human breast stromal cells treated by genotoxic agents. (**a**) Representative immunofluorescence staining (IF) images (γH2AX, left) and comparative statistics (right) of DNA damage foci (DDR) in human breast stromal cell line HBF1203. Cells were treated by DOX (doxorubicin), CIS (cisplatin) or CARB (carboplatin). DDR was profiled into 4 categories including 0 foci, 1~3 foci, 4~10 foci and > 10 foci per cell. CTRL, control. DOX, doxorubicin. CIS, cisplatin. CARB, carboplatin. DOX-treated cells were stained by IF for example. Scale bars, 15 μm. (**b**) Senescence assessment of HBF1203 cells treated by various agents used in (**a**). Cells were stained for SA-β-Gal activity 7 d after treatments. Scale bars, 15 μm. (**c**) DNA incorporation assay of HBF1203 by BrdU staining. Cells were treated by different agents as applied in (**a**) and (**b**). Scale bars, 20 μm. (**d**) Transcript expression of SPINK1 in HBF1203 cells 7 d after treatment by several agents. Signal of each treatment condition was normalized to the CTRL group. (**e**) Comparative analysis of SPINK1 transcript expression in breast stromal (HBF1203) and epithelial cells (MCF7, MDA-MB-231, MDA-MB-468, T47D and BT-474) of human breast origin. Signals normalized to untreated sample (CTRL) per cell line. (**f**) Immunoblot of SPINK1 expression in breast stromal and epithelial cells after doxorubicin (DOX) treatment as performed in (**e**). IC, intracellular extracts. CM, conditioned media. GAPDH, loading control. (**g**) Immunoblot of SPINK1 expression in PSC27 and HBF1203 lines. Native, primary stromal cell line. Vector, stromal cells lentivirally infected with a control vector. SPINK1, stromal cells lentivirally infected with the SPINK1 construct. Concentration of EGF in the CM of cells was measured by ELISA to evaluate influence of SPINK1 expression on the production of other soluble factors. (**h**) Comparative statistics of DNA damage foci (DDR) by immunofluorescence (IF) staining against

γH2AX in PSC27 and HBF1203 subline cells treated by genotoxic agents (PSC27 by BLEO, HBF1203 by DOX, to simulate the clinical conditions of PCa and BCa, respectively). DDR were classified into 4 sub-categories including 0 foci, 1~3 foci, 4~10 foci and > 10 foci per cell. (**i**) Statistics of SA-β-Gal staining results of PSC27 and HBF1203 cells treated by genotoxic agents as used in (**h**). Cells were stained 7 d after *in vitro* treatments. (**j**) Statistics of BrdU staining results of stromal cells treated by genotoxic agents as used in (**h**). (**k**) Population doubling assay of PSC27 cells grown under several different conditions. BLEO was applied to cell culture at day 22nd, and cell proliferation evaluated through day 150th. (**l**) Population doubling assessment of HBF1203 performed as described in (**k**), except that DOX was employed to damage breast stromal cells. Data are shown as mean ± SD and representative of 3 independent experiments, with 3 technical replicates run per cell-based assay. *P* values were calculated by Student's *t*-test (**b**, **c**, **d**, **e**, **i**, **j**) and two-way ANOVA (**a, h**) (^, $P > 0.05$; *, $P < 0.05$; **, $P < 0.01$; ***, $P < 0.001$).

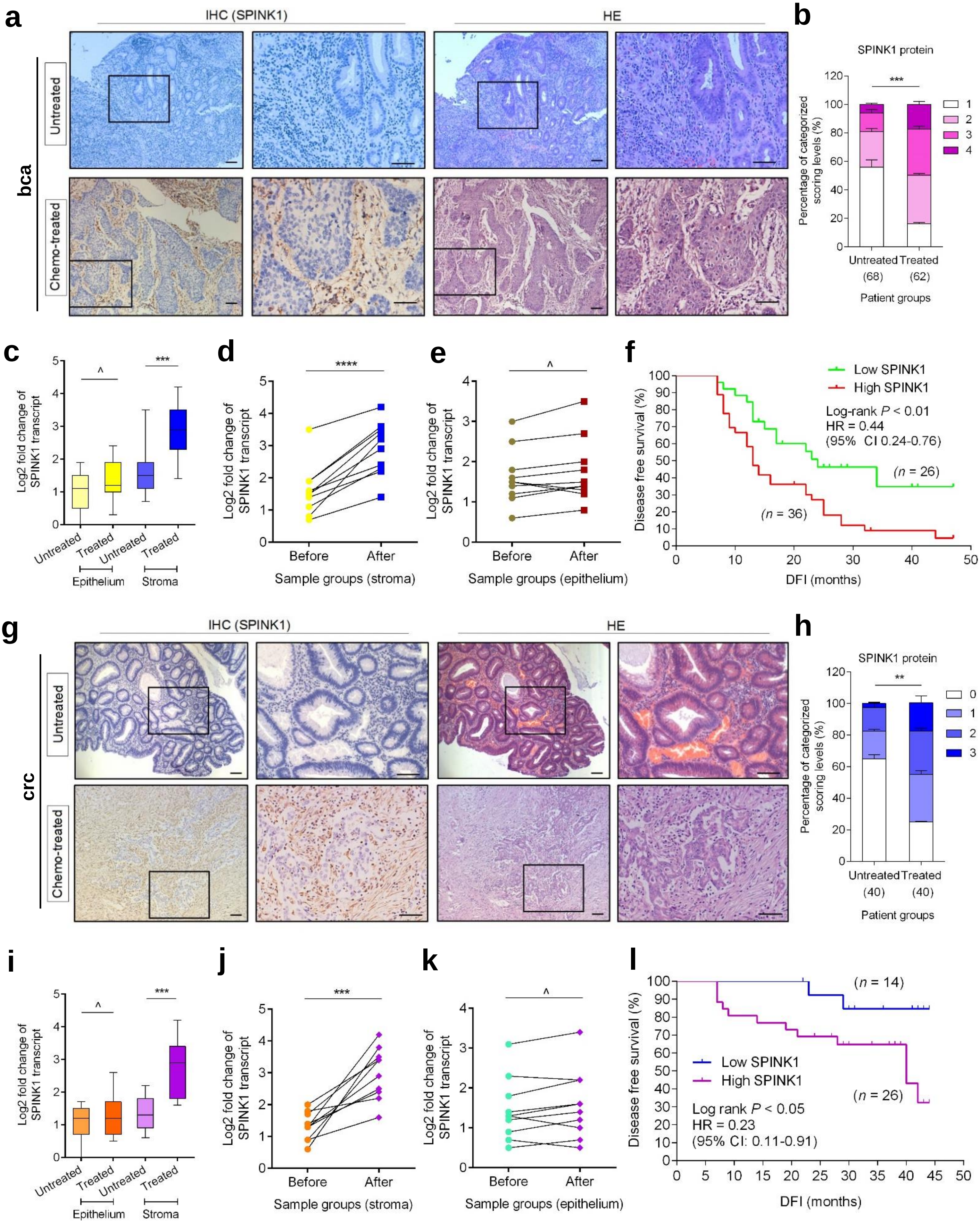

a
IHC (SPINK1)
HE
bca
Untreated
Chemo-treated
b
SPINK1 protein
Percentage of categorized scoring levels (%)
***
1
2
3
4
Untreated (68)
Treated (62)
Patient groups
c
Log2 fold change of SPINK1 transcript
^
***
Untreated
Treated
Untreated
Treated
Epithelium
Stroma
d
****
Before
After
Sample groups (stroma)
e
^
Sample groups (epithelium)
f
Low SPINK1
High SPINK1
Log-rank P < 0.01
HR = 0.44
(95% CI 0.24-0.76)
(n = 26)
(n = 36)
Disease free survival (%)
DFI (months)
g
IHC (SPINK1)
HE
crc
Untreated
Chemo-treated
h
SPINK1 protein
**
0
1
2
3
Untreated (40)
Treated (40)
Patient groups
i
^
***
Epithelium
Stroma
j
***
Sample groups (stroma)
k
^
Sample groups (epithelium)
l
(n = 14)
Low SPINK1
High SPINK1
(n = 26)
Log rank P < 0.05
HR = 0.23
(95% CI: 0.11-0.91)
Disease free survival (%)
DFI (months)

Supplementary Figure 2. SPINK1 is expressed in the tumor microenvironment (TME) of human breast cancer (BCa) and colorectal cancer (CRC) patients after chemotherapy. (**a**) Representative images of SPINK1 expression in the primary foci of human BCa patients. Left, immunohistochemical (IHC) staining. Right, hematoxylin and eosin (HE) staining. In each staining set, top tissues are from untreated patients; bottom tissues from treated. Rectangular region in the left image per staining is zoomed into the right image. Scale bars, 100 μm. (**b**) Pathological assessment of stromal SPINK1 expression in BCa patients (untreated, 68; treated, 62). Patients were pathologically assigned into 4 categories per SPINK1 staining intensity in the stroma. 1, negative; 2, weak; 3, moderate; 4, strong. Statistical comparison of untreated and treated groups is displayed. $P < 0.001$ by ANOVA. (**c**) Comparative analysis of SPINK1 expression at transcript level between different cell lineages after chemotherapy. Briefly, epithelial and stromal cells were separately acquired by laser capture microdissection (LCM) from the primary tumors of BCa patients, with RNA subsequently extracted for quantitative assessment. (**d**) Statistical appraisal of SPINK1 induction in the stroma of 10 randomly selected BCa patients. Stromal cells from both before and after treatment per same patient were isolated by LCM and subject to transcript examination. (**e**) Statistical appraisal of SPINK1 induction in the epithelium of the tumor foci of 10 randomly selected BCa patients. Cells from the same individual patients were isolated by LCM and subject to transcript examination. (**f**) Kaplan-Meier analysis of BCa patients. Disease free survival (DFS) stratified according to SPINK1 expression (low, average score < 2, green line, n = 26; high, average score ≥ 2, red line, n = 36). DFS represents the length (months) of period calculated from the date of BCa diagnosis to the point of first time disease relapse. Survival curves were plotted according to the Kaplan–Meier

method, while *P* value was calculated using a log-rank (Mantel-Cox) test. (**g**) Representative images of SPINK1 expression in the primary foci of human colorectal cancer (CRC) patients. Left, IHC staining. Right, HE staining. In each staining set, top tissues are from untreated patients; bottom tissues from treated. Rectangular region in the left image per staining is amplified into the right image. Scale bars, 100 μm. (**h**) Pathological assessment of stromal SPINK1 expression in BCa patients (untreated, 40; treated, 40). Patients were pathologically assigned into 4 categories per SPINK1 staining intensity in the stroma. 1, negative; 2, weak; 3, moderate; 4, strong. Statistical comparison of untreated and treated groups is displayed. $P < 0.01$ by ANOVA. (**i**) Comparative analysis of SPINK1 expression at transcript level between different cell lineages after chemotherapy. Stromal cells were separately acquired by LCM from the primary tumors of CRC patients, with transcripts subsequently prepared for quantitative assessment. (**j**) Induction appraisal of SPINK1 in the stroma of 10 randomly selected CRC patients. Stromal cells from both before and after treatment per patient were isolated by LCM and subject to transcript examination. (**k**) Induction appraisal of SPINK1 in the epithelium of 10 randomly selected CRC patients. Epithelial cells from both before and after treatment per patient were isolated by LCM and subject to transcript examination. (**l**) Kaplan-Meier analysis of CRC patients. Disease free survival (DFS) stratified according to SPINK1 expression (low, average score < 2, blue line, n = 14; high, average score ≥ 2, pink line, n = 26). DFS represents the length (months) of period calculated from the date of CRC diagnosis to the point of first time disease relapse. Survival curves generated according to the Kaplan–Meier method, with *P* value calculated using a log-rank (Mantel-Cox) test. Data are shown as mean ± SD and representative of 3 independent experiments. *P* values were calculated by Student's *t*-test (**c**,

d, **e**, **i**, **j**, **k**), one-way ANOVA (**b**, **h**) and log-rank test (**f**, **l**) (^, $P > 0.05$; *, $P < 0.05$; **, $P < 0.01$; ***, $P < 0.001$; ****, $P < 0.0001$). HR, hazard ratio.

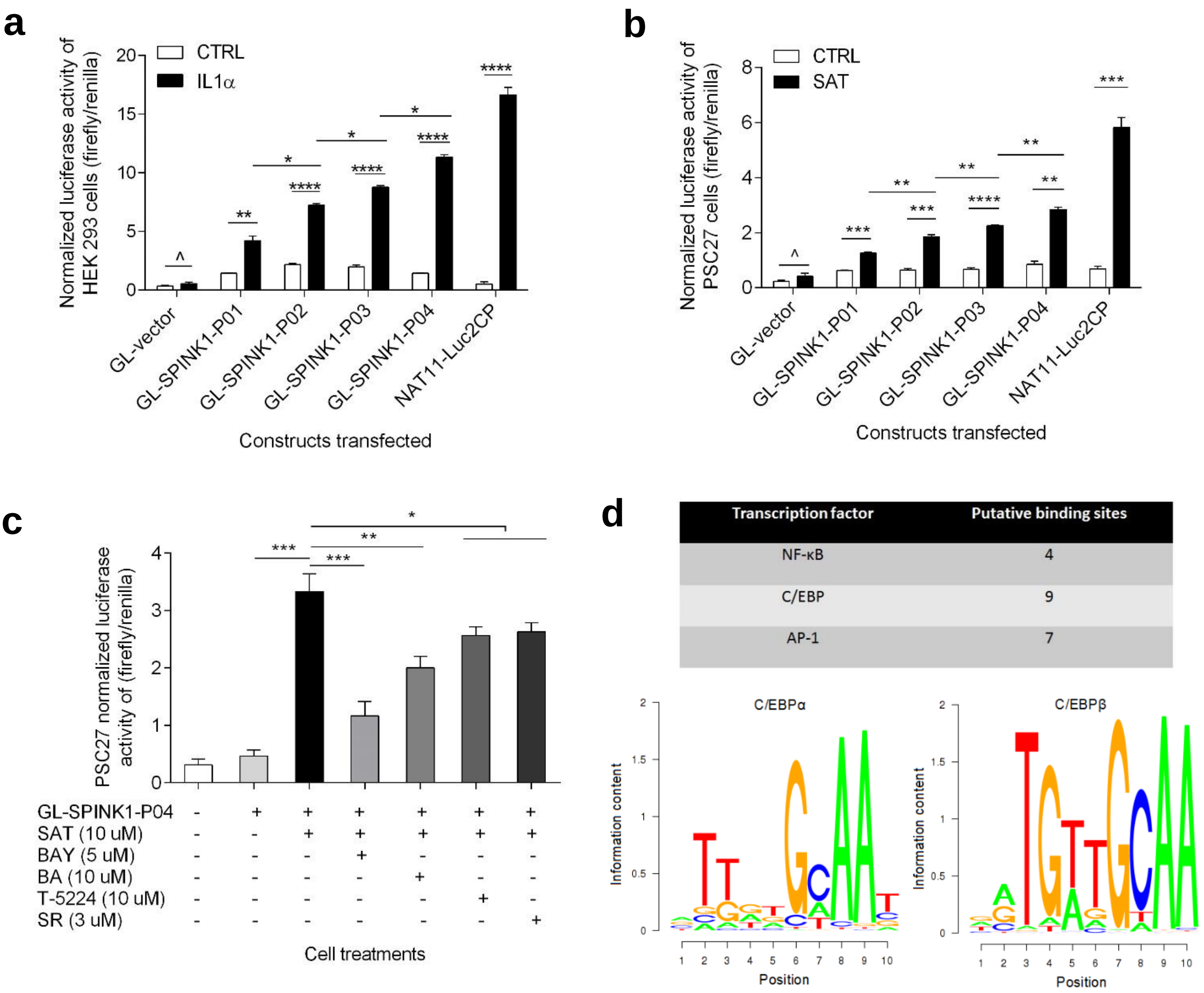

a
Normalized luciferase activity of HEK 293 cells (firefly/renilla)
CTRL
IL1α
20
15
10
5
0
^
**
*
****
*
****
*
****
****
GL-vector
GL-SPINK1-P01
GL-SPINK1-P02
GL-SPINK1-P03
GL-SPINK1-P04
NAT11-Luc2CP
Constructs transfected
b
Normalized luciferase activity of PSC27 cells (firefly/renilla)
CTRL
SAT
8
6
4
2
0
^
***
**
***
**
****
**
**
***
GL-vector
GL-SPINK1-P01
GL-SPINK1-P02
GL-SPINK1-P03
GL-SPINK1-P04
NAT11-Luc2CP
Constructs transfected
c
PSC27 normalized luciferase activity of (firefly/renilla)
4
3
2
1
0
*
**
***
***
GL-SPINK1-P04 - + + + + + +
SAT (10 uM) - - + + + + +
BAY (5 uM) - - - + - - -
BA (10 uM) - - - - + - -
T-5224 (10 uM) - - - - - + -
SR (3 uM) - - - - - - +
Cell treatments
d
Transcription factor | Putative binding sites
NF-κB | 4
C/EBP | 9
AP-1 | 7
C/EBPα
C/EBPβ
Information content
Position

Supplementary Figure 3. SPINK1 expression is mainly mediated by the NF-κB complex in response to cytokine stimulation or genotoxic stress. (**a**) Luciferase activity assay with lysates of 293T cells pre-transfected with each of GL-vector, GL-SPINK1-P01, GL-SPINK1-P02, GL-SPINK1-P03 and GL-SPINK1-P04 and subsequently treated by 10 ng/ml IL-α in culture. Data are presented as relative ratios of firefly/renilla luciferase signals. (**b**) PSC27 cells transiently transfected with the constructs used in (**a**) were treated by SAT (satraplatin, 10 μM), with cell lysates collected for luciferase assay. The NAT11-Luc2CP construct which encodes multiple NF-κB binding sites was used as a positive control. (**c**) The reporter construct pGL-SPINK1-P04 encoding the longest approximal SPINK1 promoter (4000 bp upstream of TSS) was transiently transfected into PSC27 cells before treated by SAT. BAY (Bay 11-7982, 5 μM), BA (betulinic acid, 10 μM) and T-5224 (10μM) were applied simultaneously with SAT as small molecule inhibitors against NF-κB, C/EBP family and AP-1, respectively. SR (SR 11302, 3 μM) was used as an extra control for AP-1 inhibition. Cells were lysed 7 d after treatment and subject to luciferase activity assay. Data were calculated as ratios of firefly/renilla luciferase signals. (**d**) Statistical summary of binding sites for transcription factors functionally associated with SPINK1 expression in stroma cells. Upper, putative counts provided for binding sites of NF-κB, C/EBP and AP-1 on human SPINK1 approximal promoter, with data derived from bioinformatics evaluation. Lower, representative binding motif of C/EBPα and C/EBPβ. Data are shown as mean ± SD and representative of 3 independent experiments. *P* values were calculated by Student's *t*-test (**a**, **b**, **c**) (^, $P > 0.05$; *, $P < 0.05$; **, $P < 0.01$; ***, $P < 0.001$; ****, $P < 0.0001$).

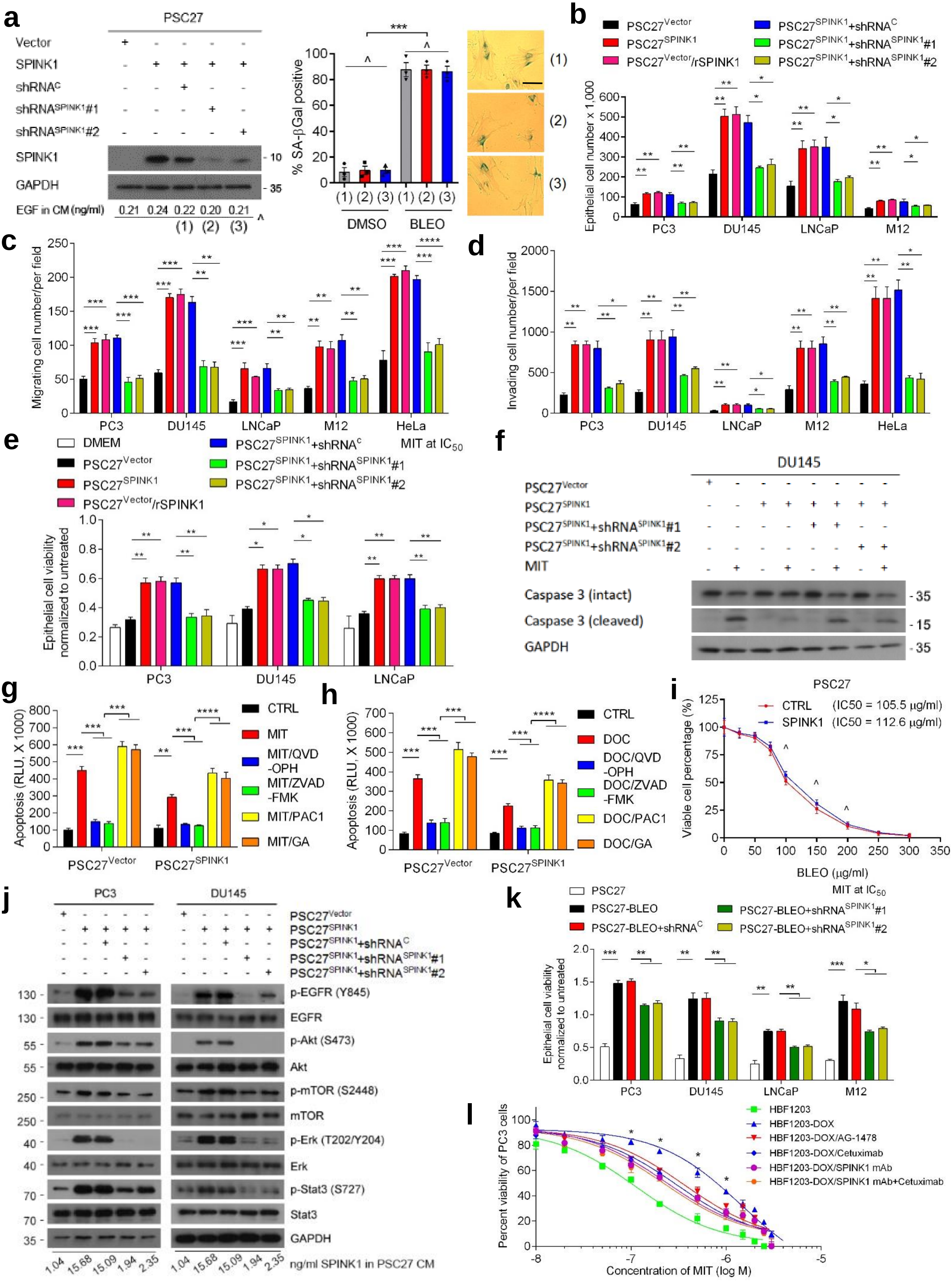
a
PSC27
Vector
SPINK1
shRNAC
shRNASPINK1#1
shRNASPINK1#2
SPINK1
GAPDH
EGF in CM (ng/ml) 0.21 0.24 0.22 0.20 0.21
(1) (2) (3)
% SA-βGal positive
DMSO
BLEO
b
PSC27Vector
PSC27SPINK1
PSC27Vector/rSPINK1
PSC27SPINK1+shRNAC
PSC27SPINK1+shRNASPINK1#1
PSC27SPINK1+shRNASPINK1#2
Epithelial cell number x 1,000
PC3 DU145 LNCaP M12
c
Migrating cell number/per field
PC3 DU145 LNCaP M12 HeLa
d
Invading cell number/per field
PC3 DU145 LNCaP M12 HeLa
e
DMEM
MIT at IC50
Epithelial cell viability normalized to untreated
PC3 DU145 LNCaP
f
DU145
PSC27Vector
PSC27SPINK1
PSC27SPINK1+shRNASPINK1#1
PSC27SPINK1+shRNASPINK1#2
MIT
Caspase 3 (intact)
Caspase 3 (cleaved)
GAPDH
g
Apoptosis (RLU, X 1000)
CTRL
MIT
MIT/QVD-OPH
MIT/ZVAD-FMK
MIT/PAC1
MIT/GA
PSC27Vector PSC27SPINK1
h
CTRL
DOC
DOC/QVD-OPH
DOC/ZVAD-FMK
DOC/PAC1
DOC/GA
i
PSC27
CTRL (IC50 = 105.5 μg/ml)
SPINK1 (IC50 = 112.6 μg/ml)
Viable cell percentage (%)
BLEO (μg/ml)
j
PC3
DU145
p-EGFR (Y845)
EGFR
p-Akt (S473)
Akt
p-mTOR (S2448)
mTOR
p-Erk (T202/Y204)
Erk
p-Stat3 (S727)
Stat3
GAPDH
1.04 15.68 15.09 1.94 2.35 1.04 15.68 15.09 1.94 2.35 ng/ml SPINK1 in PSC27 CM
k
PSC27
PSC27-BLEO
PSC27-BLEO+shRNAC
PSC27-BLEO+shRNASPINK1#1
PSC27-BLEO+shRNASPINK1#2
MIT at IC50
Epithelial cell viability normalized to untreated
PC3 DU145 LNCaP M12
l
HBF1203
HBF1203-DOX
HBF1203-DOX/AG-1478
HBF1203-DOX/Cetuximab
HBF1203-DOX/SPINK1 mAb
HBF1203-DOX/SPINK1 mAb+Cetuximab
Percent viability of PC3 cells
Concentration of MIT (log M)

Supplementary Figure 4. Stromal cell-derived SPINK1 changes the phenotypes of cancer cells. (a) Immunoblot examination of SPINK1 expressed in PSC27 sublines established with different lentivirual constructs. Cells were infected with lentiviral particles encoding either the control vector or full length human SPINK1 sequence. Stable PSC27-SPINK1 cells were subsequently transduced with scramble or SPINK1-specific shRNAs. GAPDH, loading control for protein lysates. Bottom, EGF concentration in the CM was measured by ELISA to determine the influence of SPINK1 knockdown in stromal cells. Right, statistical profiling of SA-β-Gal staining results of PSC27 sublines after BLEO treatment. DMSO, vehicle control. (**b**) PCa cell lines including PC3, DU145, LNCaP and M12 were treated with the conditioned media (CM) from PSC27 cells for 3 days, and subject to cell proliferation assay. $PSC27^{Vector}$ was used as control for cells exogenously expressing SPINK1 ($PSC27^{SPINK1}$). Scramble or SPINK-specific shRNAs were used to transduce $PSC27^{SPINK1}$ cells to generate stable sublines ($PSC27^{SPINK1}$+$shRNA^{C}$, $PSC27^{SPINK1}$+$PSC27^{SPINK1}$ #1 and $PSC27^{SPINK1}$+$PSC27^{SPINK1}$#2). (**c**) Migration assessment of PCa cells after cultured for 3 days in the CM from sublines of PSC27. HeLa cells were examined as a positive control. (**d**) Invasion assay of PCa cells across the basement membrane of transwells upon culture with the CM from sublines of PSC27 for 3 days. HeLa line served as a positive control. (**e**) Chemoresistance assay of PCa cells upon culture with the CM from PSC27 sublines. MIT (mitoxantrone) was applied at the concentration of IC50 value pre-determined per cell line. (**f**) Immunoblot analysis of protein lysates prepared from DU145, cultured with the CM of PSC27 sublines for consecutive 3 days in the presence or absence of MIT. Intact and cleaved caspase 3 was probed. GAPDH, loading control. (**g**) Apoptotic assay for combined activities of caspase 3/7 determined 24 h after exposure of PC3

cells to CM of stromal cells while being treated by mitoxantrone (MIT) in the presence of absence of caspase inhibitors including QVD-OPH and ZVAD-FMK, or caspase activators including PAC1 and gambogic acid (GA). RLU, relative luciferase unit. (**h**) Apoptotic assay performed in the way similar as the one in (**g**), except that PAC (paclitaxel) was applied instead of MIT. RLU, relative luciferase unit. (**i**) Survival curves of PSC27 sublines transduced with either empty vector (CTRL) or a SPINK1 construct (SPINK1). Cells were exposed to increasing concentrations of BLEO (25-300 µg/ml), and the number of surviving cells after 3-day consecutive culture was counted and normalized to the untreated control, with the IC50 value calculated per line. (**j**) Immunoblot analysis of EGFR-associated pathways in PC3 and DU145 cells treated by the CM from PSC27 sublines as examined in (**a**). Antibodies of p-EGFR (Y845), p-Akt (S473), p-mTOR (S2448), p-Erk (T202/Y204) and p-Stat3 (S727) were used, with the total protein per molecule and GAPDH probed as loading control. (**k**) Chemoresistance assay of PCa cells cultured with the CM from PSC27 sublines analyzed in (**a**) after treatment by BLEO. MIT was applied at the concentration of IC50 value pre-determined per cell line. (**l**) Dose-response curves (non-linear regression/curve fit) plotted from drug-based survival assays of MDA-MB-231 cells cultured with the CM of HBF1203 native or damaged by doxorubicin (HBF1203-DOX), and concurrently treated by a wide range of concentrations MIT. AG-1478 (2 µM), cetuximab (50 µg/ml) or SPINK1 mAb (1 µg/ml) were applied with PSC27 CM. Data are shown as mean ± SD and representative of 3 independent experiments. *P* values were calculated by Student's *t*-test (**a**, **b**, **c**, **d**, **e**, **g**, **h**, **i**, **k**) (^, $P > 0.05$. *, $P < 0.05$. **, $P < 0.01$. ***, $P < 0.001$. ****, $P < 0.0001$).

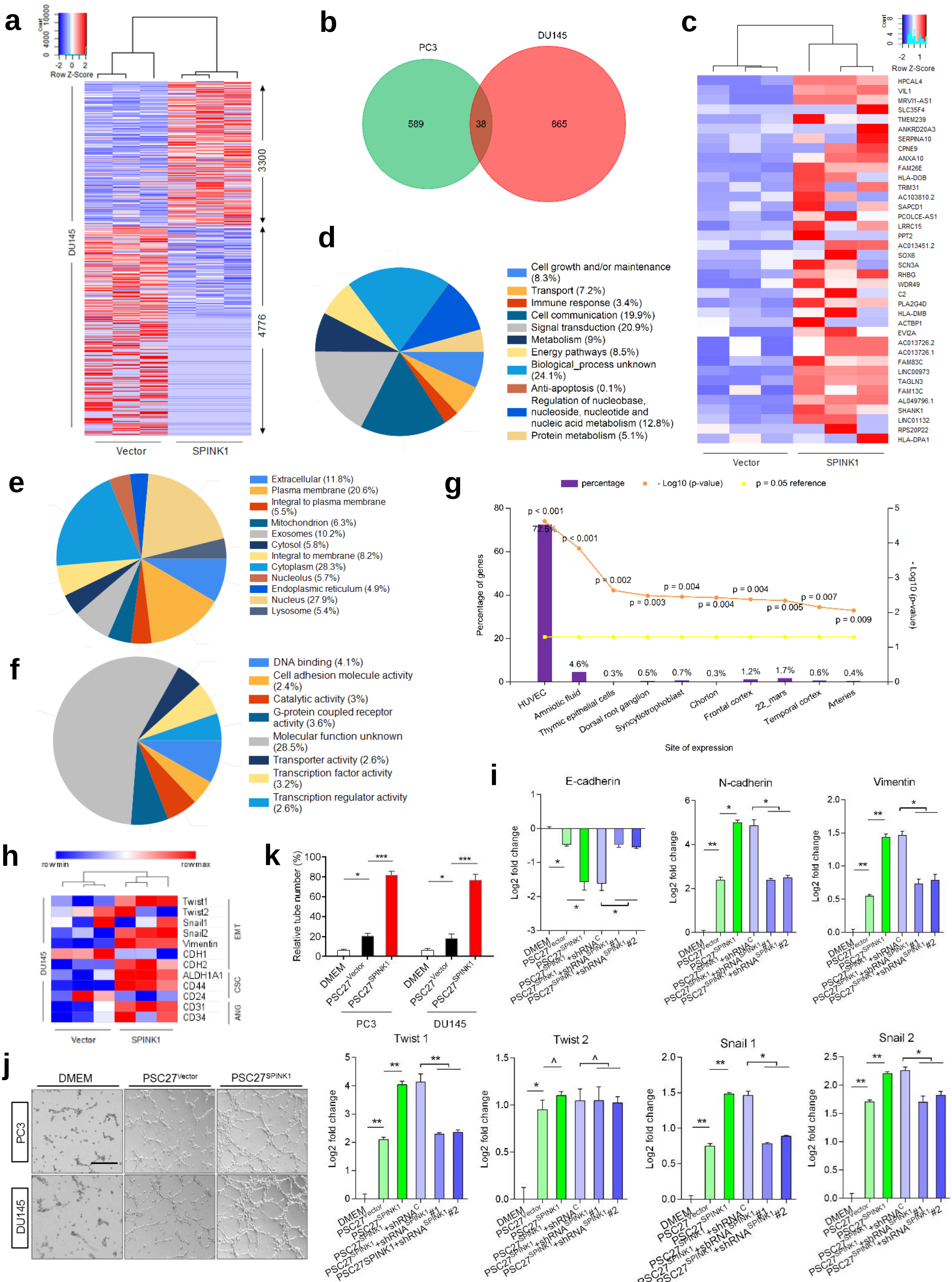

a
Row Z-Score
DU145
3300
4776
Vector
SPINK1
b
PC3
DU145
589
38
865
c
Row Z-Score
HPCAL4
VIL1
MRVI1-AS1
SLC35F4
TMEM239
ANKRD20A3
SERPINA10
CPNE9
ANXA10
FAM26E
HLA-DOB
TRIM31
AC103810.2
SAPCD1
PCOLCE-AS1
LRRC15
PPT2
AC013451.2
SOX6
SCN3A
RHBG
WDR49
C2
PLA2G4D
HLA-DMB
ACTBP1
EVI2A
AC013726.2
AC013726.1
FAM83C
LINC00973
TAGLN3
FAM13C
AL049796.1
SHANK1
LINC01132
RPS20P22
HLA-DPA1
Vector
SPINK1
d
Cell growth and/or maintenance (8.3%)
Transport (7.2%)
Immune response (3.4%)
Cell communication (19.9%)
Signal transduction (20.9%)
Metabolism (9%)
Energy pathways (8.5%)
Biological_process unknown (24.1%)
Anti-apoptosis (0.1%)
Regulation of nucleobase, nucleoside, nucleotide and nucleic acid metabolism (12.8%)
Protein metabolism (5.1%)
e
Extracellular (11.8%)
Plasma membrane (20.6%)
Integral to plasma membrane (5.5%)
Mitochondrion (6.3%)
Exosomes (10.2%)
Cytosol (5.8%)
Integral to membrane (8.2%)
Cytoplasm (28.3%)
Nucleolus (5.7%)
Endoplasmic reticulum (4.9%)
Nucleus (27.9%)
Lysosome (5.4%)
f
DNA binding (4.1%)
Cell adhesion molecule activity (2.4%)
Catalytic activity (3%)
G-protein coupled receptor activity (3.6%)
Molecular function unknown (28.5%)
Transporter activity (2.6%)
Transcription factor activity (3.2%)
Transcription regulator activity (2.6%)
g
percentage
- Log10 (p-value)
p = 0.05 reference
Percentage of genes
- Log10 (p-value)
p < 0.001
72.5%
p < 0.001
p = 0.002
p = 0.003
p = 0.004
p = 0.004
p = 0.004
p = 0.005
p = 0.007
p = 0.009
4.6%
0.3%
0.5%
0.7%
0.3%
1.2%
1.7%
0.6%
0.4%
HUVEC
Amniotic fluid
Thymic epithelial cells
Dorsal root ganglion
Syncytiotrophoblast
Chorion
Frontal cortex
22_mars
Temporal cortex
Arteries
Site of expression
h
row min
row max
Twist1
Twist2
Snail1
Snail2
Vimentin
CDH1
CDH2
ALDH1A1
CD44
CD24
CD31
CD34
EMT
CSC
ANG
DU145
Vector
SPINK1
i
E-cadherin
N-cadherin
Vimentin
Log2 fold change
DMEM
PSC27Vector
PSC27SPINK1
PSC27SPINK1+shRNA C
PSC27SPINK1+shRNA SPINK1 #1
PSC27SPINK1+shRNA SPINK1 #2
j
DMEM
PSC27Vector
PSC27SPINK1
PC3
DU145
Twist 1
Twist 2
Snail 1
Snail 2
k
Relative tube number (%)
DMEM
PSC27Vector
PSC27SPINK1
PC3
DU145

Supplementary Figure 5. Transcriptomic-wide expression of prostate cancer (PCa) cells is modified by stromal cell-derived SPINK1. (**a**) Heatmap showing differentially expressed human transcripts in DU145 cells after consecutive 3-day culture with SPINK1-containing CM collected from PSC27 stromal cells. In contrast to cancer cells cultured with control media (vector), there were 3300 and 4776 genes upregulated and downregulated, respectively, in those treated with conditioned media from SPINK1-expressing PSC27 cells (SPINK1). (**b**) Venn diagram presenting the overlap of 38 transcripts upregulated in PC3 and DU145 cells upon treatment with SPINK1-containing CM from stromal cells (627 and 903 genes were significantly upregulated with average fold change ≥ 4 after SPINK1-CM treatment of PC3 and DU145, respectively). (**c**) Heatmap depicting the top 38 co-upregulated transcripts by both PC3 and DU145 cells. Genes sorted according to their expression fold changes in DU145 cells. (**d**) GO-pie chart displaying the biological process of the 3300 transcripts upregulated in DU145 after SPINK1-CM treatment. (**e**) GO-pie chart manifesting the cellular component of the 3300 transcripts upregulated in DU145 after SPINK1 stimulation. (**f**) GO-pie chart showing the molecular function of the 3300 DU145 transcripts analyzed in (**d**) and (**e**). (**g**) Column chart depicting the sites of expression of 3300 transcripts upregulated in DU145 cells after SPINK1 stimulation, with percentage and log10 (*P* value) per specific site indicated on the left and right Y axis, respectively. (**h**) Heatmap of gene expression signatures associated with phenotypic changes, namely EMT/CSC/ANG, after SPINK1 stimulation of DU145 cells *in vitro.* EMT, epithelial-to-mesenchymal transition; CSC, cancer stem cell; ANG, angiogenesis. Data derived from qRT-PCR assays. (**i**) Quantitative RT-PCR analysis of the transcript expression of EMT-specific markers E-cadherin, N-cadherin and vimentin, as well as EMT regulators Twist 1, Twist

2, Snail 1 and Snail 2 in DU145 cells treated with the CM of PSC27 sublines. Data per factor were normalized to DMEM-cultured cells. (**j**) *In vitro* tube formation assay to measure the angiogenesis-inducing capacity of stromal SPINK1. PCa cells (PC3 and DU145) were seeded on top of a basement membrane matrigel layer and incubated in the CM of PSC27 sublines for 10 h. Cells were stained with calcein-AM and photographed by phase contrast microscopy. Scale bars, 100 μm. (**k**) Statistical assessment of the capacity of tube formation by PCa cells cultured on the top of polymerized matrigel in 12-well plates loaded with different CM of PSC27 cells. Tubular structures were quantified by manual counting of high power fields (HPFs). Data are shown as mean ± SD and representative of 3 independent experiments. *P* values were calculated by Student's *t*-test (**i**) (^, $P > 0.05$; *, $P < 0.05$; **, $P < 0.01$).

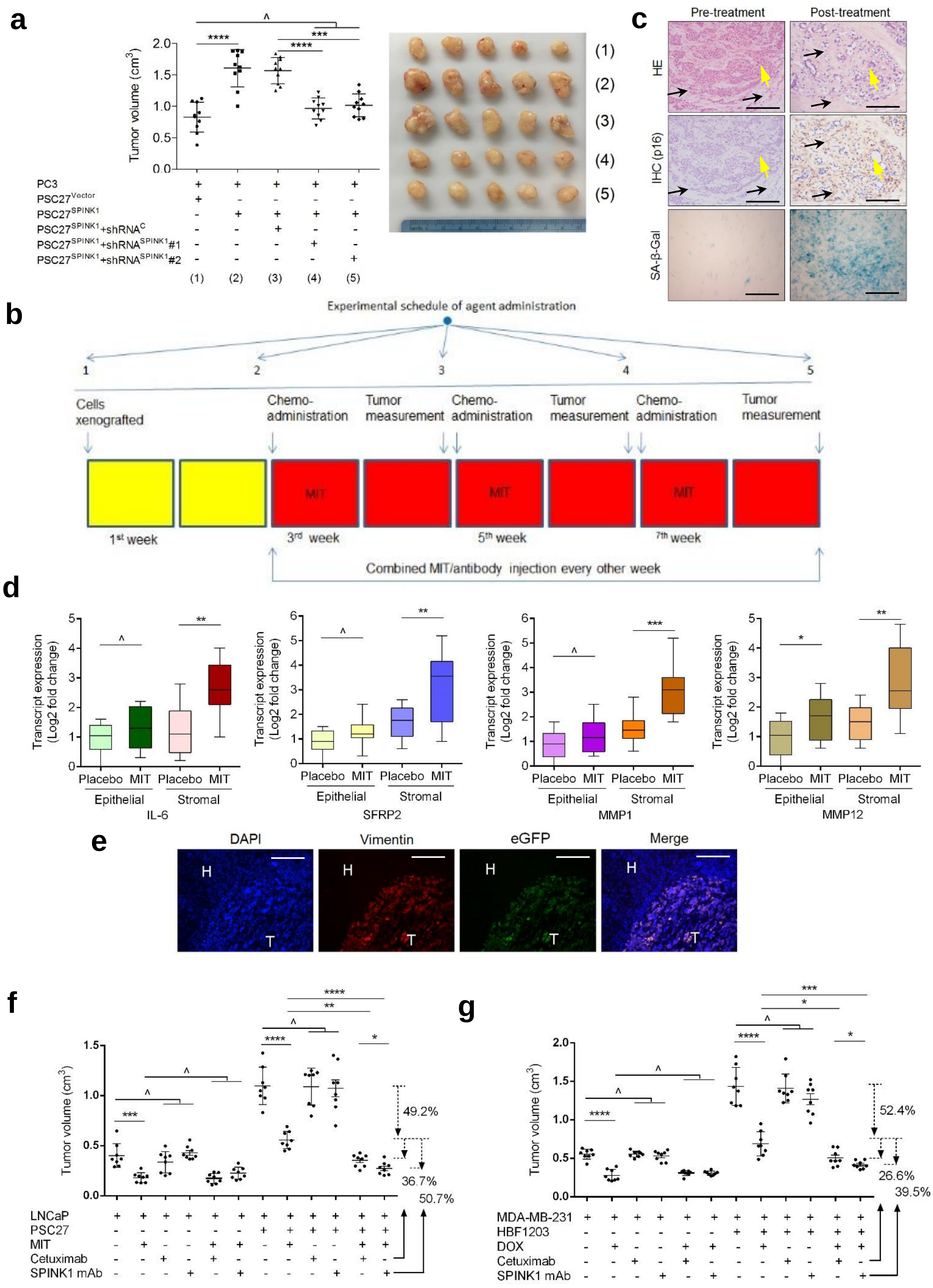
a
Tumor volume (cm3)
2.0
1.5
1.0
0.5
0.0
****
^
***
****
PC3
PSC27Vector
PSC27SPINK1
PSC27SPINK1+shRNAC
PSC27SPINK1+shRNASPINK1#1
PSC27SPINK1+shRNASPINK1#2
(1) (2) (3) (4) (5)
c
Pre-treatment
Post-treatment
HE
IHC (p16)
SA-β-Gal
b
Experimental schedule of agent administration
1 2 3 4 5
Cells xenografted
Chemo-administration
Tumor measurement
Chemo-administration
Tumor measurement
Chemo-administration
Tumor measurement
MIT
MIT
MIT
1st week
3rd week
5th week
7th week
Combined MIT/antibody injection every other week
d
Transcript expression (Log2 fold change)
Placebo MIT Placebo MIT
Epithelial Stromal
IL-6
SFRP2
MMP1
MMP12
e
DAPI
Vimentin
eGFP
Merge
H
T
f
Tumor volume (cm3)
1.5
1.0
0.5
0.0
49.2%
36.7%
50.7%
LNCaP
PSC27
MIT
Cetuximab
SPINK1 mAb
g
Tumor volume (cm3)
2.0
1.5
1.0
0.5
0.0
52.4%
26.6%
39.5%
MDA-MB-231
HBF1203
DOX
Cetuximab
SPINK1 mAb

Supplementary Figure 6. Paracrine SPINK1 confers therapeutic resistance on solid tumors developed in experimental animals. (**a**) Statistics of tumor volume measured at the end of an 8-week growth period. PC3 cells were xenografted alone or together with PSC27 cells to the hind flank of SCID mice. Prior to implantation, PSC27 cells were transduced with the control vector or SPINK1 construct to make stable sublines ($PSC27^{Vector}$, and $PSC27^{SPINK1}$, respectively). $PSC27^{SPINK1}$ cells were subsequently subject to lentiviral infection to make SPINK1-null sublines ($PSC27^{SPINK1}$+$shRNA^{SPINK1}$#1 and $PSC27^{SPINK1}$+$shRNA^{SPINK1}$#2), with scramble shRNA used as non-specific control ($PSC27^{SPINK1}$+$shRNA^{C}$). Right, representative images for tumors isolated from SCID mice, with numeric numbers corresponding to those provided in the left graph. (**b**) Strategic design of drug administration and tumor surveillance for preclinical trial. PC3 cells alone or combined with PSC27 cells were inoculated subcutaneously to SCID mice 2 weeks prior to the initiation of chemotherapy. The chemotherapeutic agent MIT was provided on the first day of each week starting from the 3rd week, then given every other week with a total number of 3 doses. Therapeutic antibodies (cetuximab or SPINK1 mAb) were given 12 h before each time of MIT delivery (totally 3 doses in the regimen). At the end of 8 weeks mice were sacrificed with tumor volume measured, and histologically analyzed. (**c**) Representative images of *in vivo* cellular senescence after MIT-mediated chemotherapy. PC3/PSC27 tumors were collected at the end of therapeutic regimen and subject to histological assessment. Images from staining of HE, IHC (anti-p16 as primary antibody) and SA-β-gal staining are acquired for comparative analysis. Black arrows, stromal cells. Yellow arrowheads, cancer cells. Scale bars, 150 μm. (**d**) Transcript analysis of several canonical SASP factors including IL-6, SFRP2, MMP1 and MMP12 expressed in stromal cells isolated from the tumors

of SCID mice. Tissues from animals implanted with both stromal and cancer cells in tumor grafts were subject to LCM isolation, RNA preparation and qRT-PCR assays. (**e**) Immunofluorescence assessment of tumor xenografts. Tumor sections were subject to staining with an antibody against vimentin. Blue, DAPI; red, vimentin; green, signals from PSC27 cells pre-transduced with a marker vector encoding enhanced green fluorescent protein (eGFP). H, host; T, tumor. Scale bars, 150 μm. (**f**) Statistical comparison of tumor growth in animals after treatment by different agents. Mice received LNCaP cells implanted alone or combined with PSC27 cells, before treated by the chemotherapeutic drug (MIT) alone or combined with cetuximab or SPINK1 mAB. Tumor volumes were measured at the end of an 8-week preclinical regimen. (**g**) A similar statistical comparison performed for tumors grown in mice as depicted in (**f**), except that animals received breast cancer cells MDA-MB-231 alone or combined with breast stromal cells HBF12037. Mice were treated by the chemotherapeutic drug DOX only or together with monoclonal antibodies (cetuximab or SPINK1 mAb). Tumor volumes were measured at the end of an 8-week preclinical regimen. Data are shown as mean ± SD and representative of 3 independent experiments. n = 10 per treatment arm. MIT, mitoxantrone. DOX, doxorubicin. *P* values were calculated by Student's *t*-test (**a**, **d**, **f**, **g**) (^, $P > 0.05$; *$P < 0.05$; **$P < 0.01$; ***$P < 0.001$; ****$P < 0.0001$).

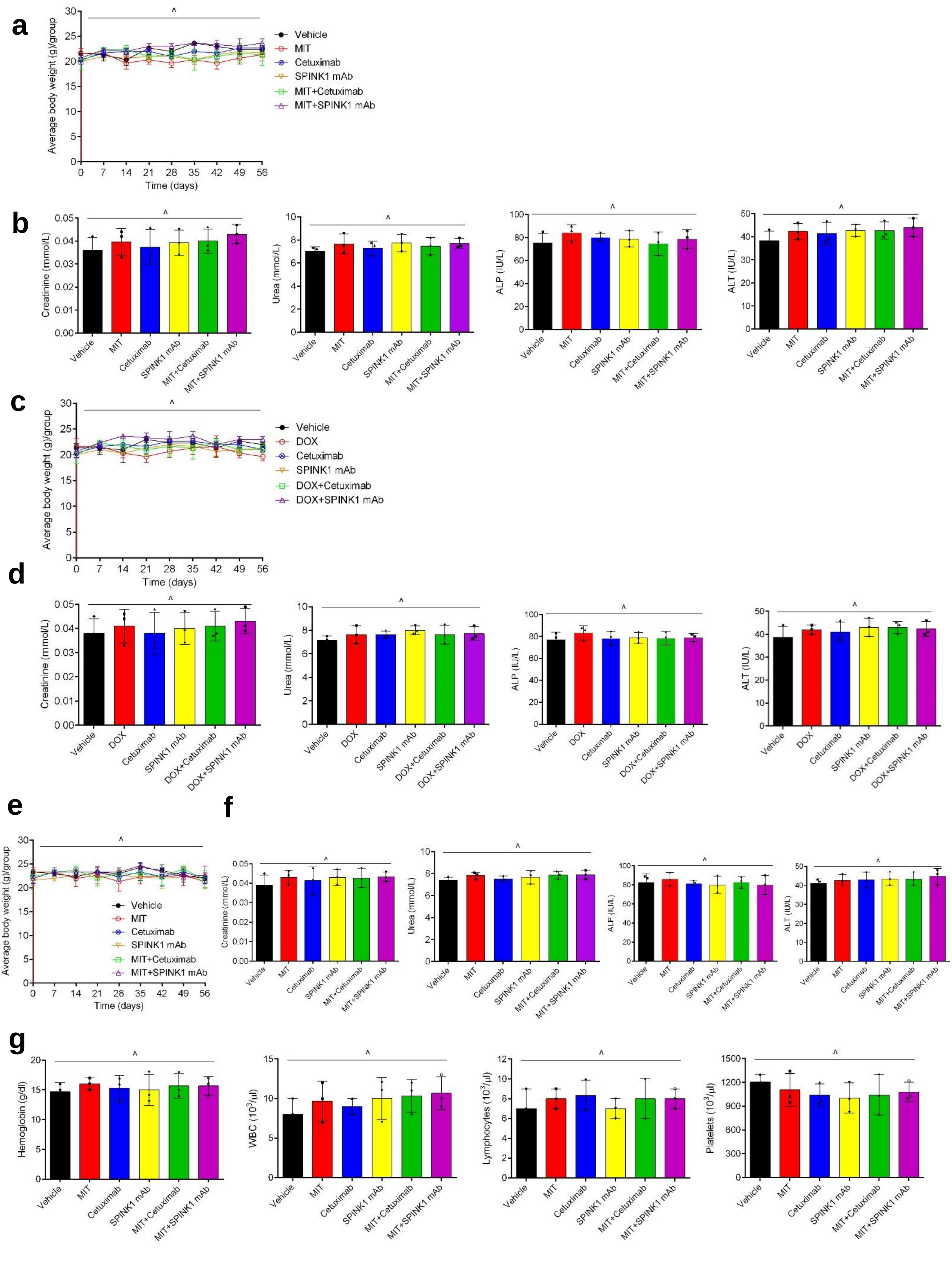

a
Average body weight (g)/group
Time (days)
^
Vehicle
MIT
Cetuximab
SPINK1 mAb
MIT+Cetuximab
MIT+SPINK1 mAb
b
Creatinine (mmol/L)
Urea (mmol/L)
ALP (IU/L)
ALT (IU/L)
Vehicle
MIT
Cetuximab
SPINK1 mAb
MIT+Cetuximab
MIT+SPINK1 mAb
c
Average body weight (g)/group
Time (days)
Vehicle
DOX
Cetuximab
SPINK1 mAb
DOX+Cetuximab
DOX+SPINK1 mAb
d
Creatinine (mmol/L)
Urea (mmol/L)
ALP (IU/L)
ALT (IU/L)
Vehicle
DOX
Cetuximab
SPINK1 mAb
DOX+Cetuximab
DOX+SPINK1 mAb
e
Average body weight (g)/group
Time (days)
Vehicle
MIT
Cetuximab
SPINK1 mAb
MIT+Cetuximab
MIT+SPINK1 mAb
f
Creatinine (mmol/L)
Urea (mmol/L)
ALP (IU/L)
ALT (IU/L)
g
Hemoglobin (g/dl)
WBC (10^3/μl)
Lymphocytes (10^3/μl)
Platelets (10^3/μl)
Vehicle
MIT
Cetuximab
SPINK1 mAb
MIT+Cetuximab
MIT+SPINK1 mAb

Supplementary Figure 7. Chemotherapeutic and/or targeting agents generate negligible effects on body weight, biochemistry, and blood counts of experimental mice. (**a**) Mouse body weights were determined once a week until the end of the therapeutic regimen. Chemotherapeutic agent MIT (0.2 mg/kg) was administered alone or with the targeting antibody (cetuximab or SPINK1 mAb, each 10.0 mg/kg) on 1st day of week 3, 5 and 7 after tumor implantation (PC3/PSC27) to SCID mice. (**b**) Terminal bleeds were taken via cardiac punctures on day 56. Serum levels of creatinine, urea, alkaline phosphatase (ALP) and alanine aminotransferase (ALT) were analyzed for toxicity appraisal with SCID mice developing prostate tumors. (**c**) Mouse body weights were determined as described in a strategy similar to (**a**). Chemotherapeutic agent DOX (1.0 mg/kg) was administered alone or with the targeting antibody (cetuximab or SPINK1 mAb, each 10.0 mg/kg) on 1st day of week 3, 5 and 7 after tumor implantation (MDA-MB-231/HBF1203) to SCID mice. (**d**) Terminal bleeds were taken via cardiac punctures on the last day of the 8-week regimen. Serum levels of creatinine, urea, alkaline phosphatase (ALP) and alanine aminotransferase (ALT) were analyzed for toxicity appraisal with SCID mice developing breast tumors. (**e**) Mouse body weights were determined once a week until the end of a therapeutic regimen applied to immunocompetent animals. Chemotherapeutic agent MIT (0.2 mg/kg) was administered alone or with the targeting antibody (cetuximab or SPINK1 mAb, each 10.0 mg/kg) on 1st day of week 3, 5 and 7 to wild type C57BL/6 mice. (**f**) Terminal bleeds were taken via cardiac punctures on the last day of the regime. Serum levels of creatinine, urea, alkaline phosphatase (ALP) and alanine aminotransferase (ALT) were analyzed for potential toxicity to C57BL/6 mice. (**g**) Blood counts were measured to evaluate potential effect of therapeutic agents on the immune system and

tissue homeostasis of C57BL/6. WBC, white blood count. Data are shown as mean ± SD and representative of 3 independent experiments. n = 3 per treatment arm. MIT. *P* values were calculated by Student's *t*-test (**a-g**) (^, $P > 0.05$).

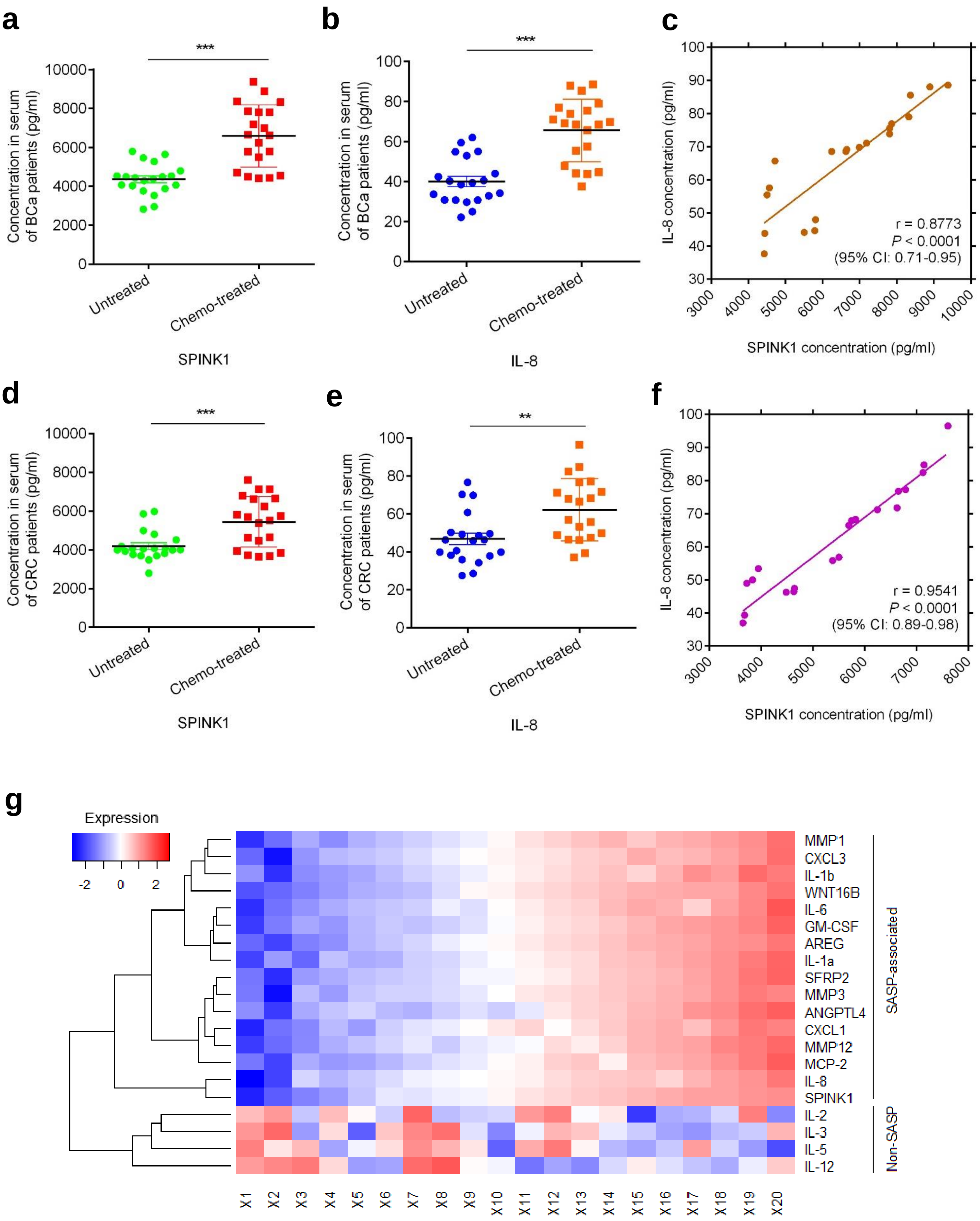

a
***
Concentration in serum of BCa patients (pg/ml)
Untreated
Chemo-treated
SPINK1
b
***
Concentration in serum of BCa patients (pg/ml)
Untreated
Chemo-treated
IL-8
c
IL-8 concentration (pg/ml)
r = 0.8773
P < 0.0001
(95% CI: 0.71-0.95)
SPINK1 concentration (pg/ml)
d
***
Concentration in serum of CRC patients (pg/ml)
Untreated
Chemo-treated
SPINK1
e
**
Concentration in serum of CRC patients (pg/ml)
Untreated
Chemo-treated
IL-8
f
IL-8 concentration (pg/ml)
r = 0.9541
P < 0.0001
(95% CI: 0.89-0.98)
SPINK1 concentration (pg/ml)
g
Expression
MMP1
CXCL3
IL-1b
WNT16B
IL-6
GM-CSF
AREG
IL-1a
SFRP2
MMP3
ANGPTL4
CXCL1
MMP12
MCP-2
IL-8
SPINK1
IL-2
IL-3
IL-5
IL-12
SASP-associated
Non-SASP

Supplementary Figure 8. SPINK1 is a novel biomarker that indicates the SASP development *in vivo* and predicts adverse outcome in the post-treatment stage of cancer patients. (**a**) Abundance of SPINK1 protein in the serum of untreated and chemo-treated BCa patients. Data were derived from ELISA measurement, n = 20. (**b**) Abundance of IL-8 protein in patient serum analyzed in (**a**), data from ELISA assays, n = 20. (**c**) Scatterplot showing correlation between SPINK1 and IL-8 in the serum of individual BCa patients studied in (**a**) and (**b**). Pearson's correlation coefficient, *P* value and confidence interval are indicated in the plot, n = 20. (**d**) Abundance of SPINK1 protein in the serum of untreated and chemo-treated CRC patients. Data were derived from ELISA measurement, n = 20. (**e**) Abundance of IL-8 protein in patient serum analyzed in (**d**), data from ELISA assays, n = 20. (**f**) Scatterplot showing correlation between SPINK1 and IL-8 in the serum of individual CRC patients studied in (**d**) and (**e**). Pearson's correlation coefficient, *P* value and confidence interval are indicated in the plot, n = 20. (**g**) Heatmap showing the relative expression of a panel of SASP signature factors in the tumor stroma of PCa patients, and the correlation of SPINK1/IL-8 with these factors (n = 20). Stromal cells in the tumor tissues were isolated via LCM and expression of each target factor was measured by qRT-PCR, with signals per factor group normalized to that of the sample showing the lowest expression value. A subset of inflammatory factors typically not considered as SASP components was examined as random control, including IL-2/3/5/12. Correlations of these factors are presented as dendrogram organized by hierarchical clustering. Data are shown as mean ± SD and representative of 3 independent experiments. *P* values were calculated by Student's *t*-test (**a**, **b**, **d**, **e**) and Pearson analysis (**c**, **f**) (***P* < 0.01; ****P* < 0.001).

Fig. 1f

SPINK1 (IC)

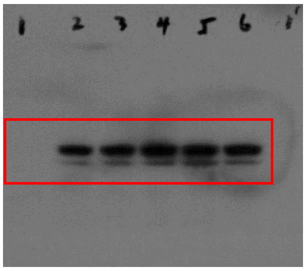

- 10

SPINK1 (CM)

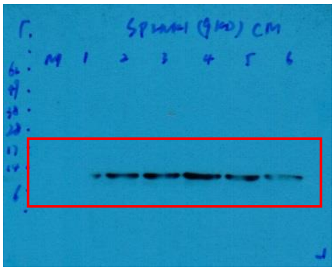

- 10

GAPDH

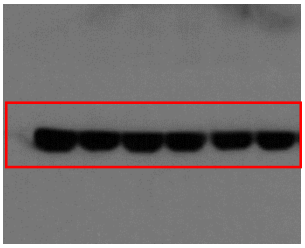

- 35

Fig. 1i

SPINK1 (IC)

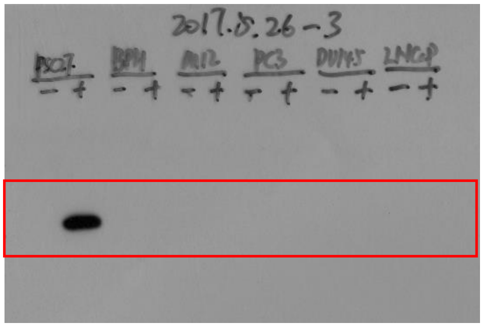

- 10

SPINK1 (CM)

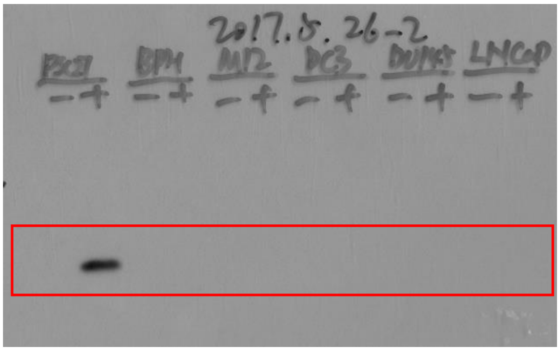

- 10

GAPDH

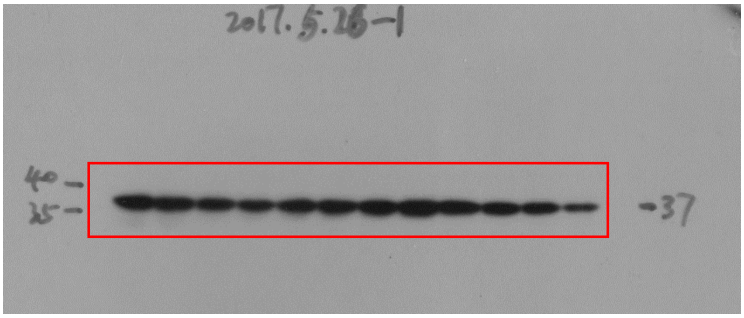

- 35

Supplementary Figure 9. The uncropped scans of immunoblots

Fig. 4a

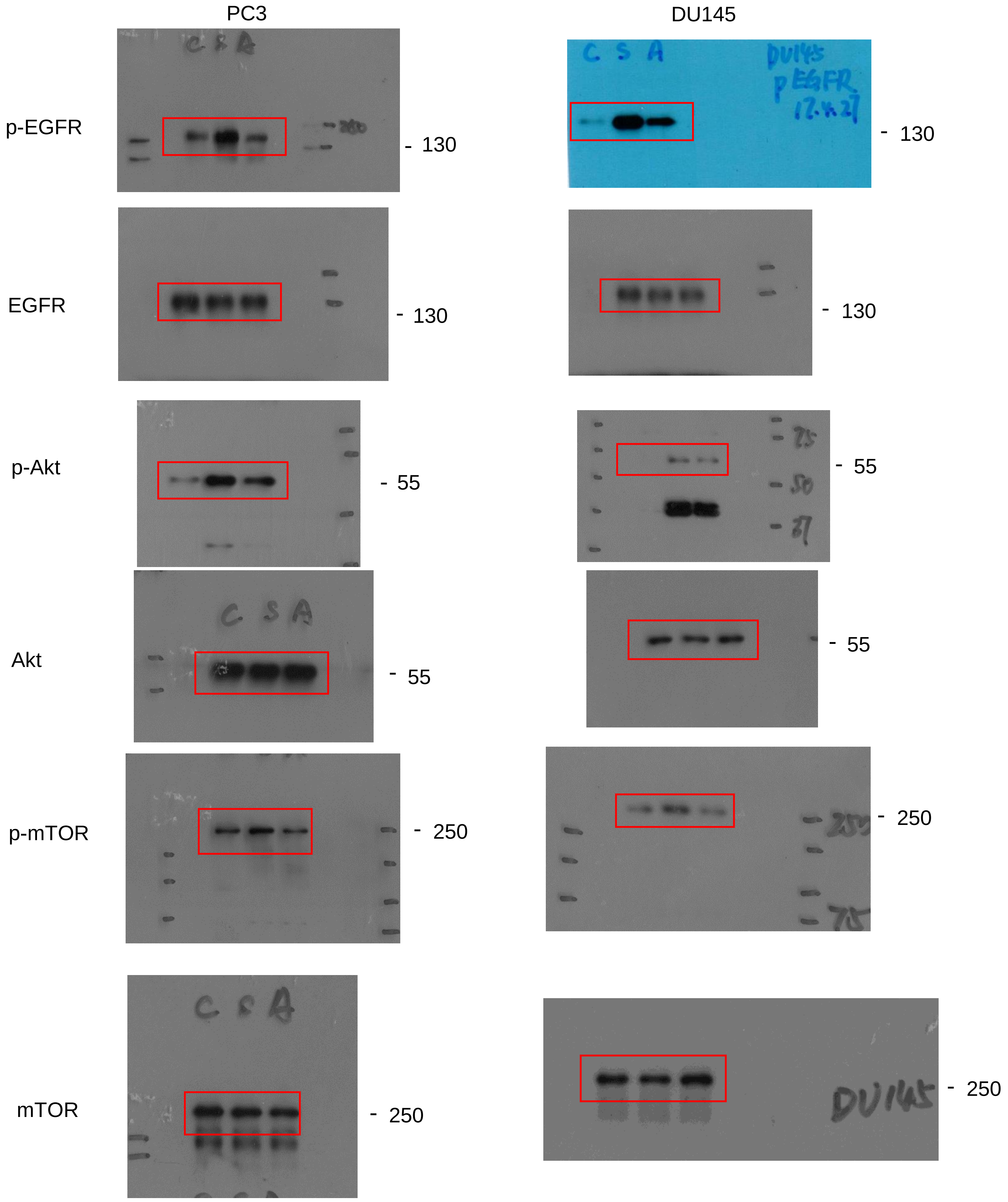

Supplementary Figure 9. The uncropped scans of immunoblots (continued)

Fig. 4a (continued)

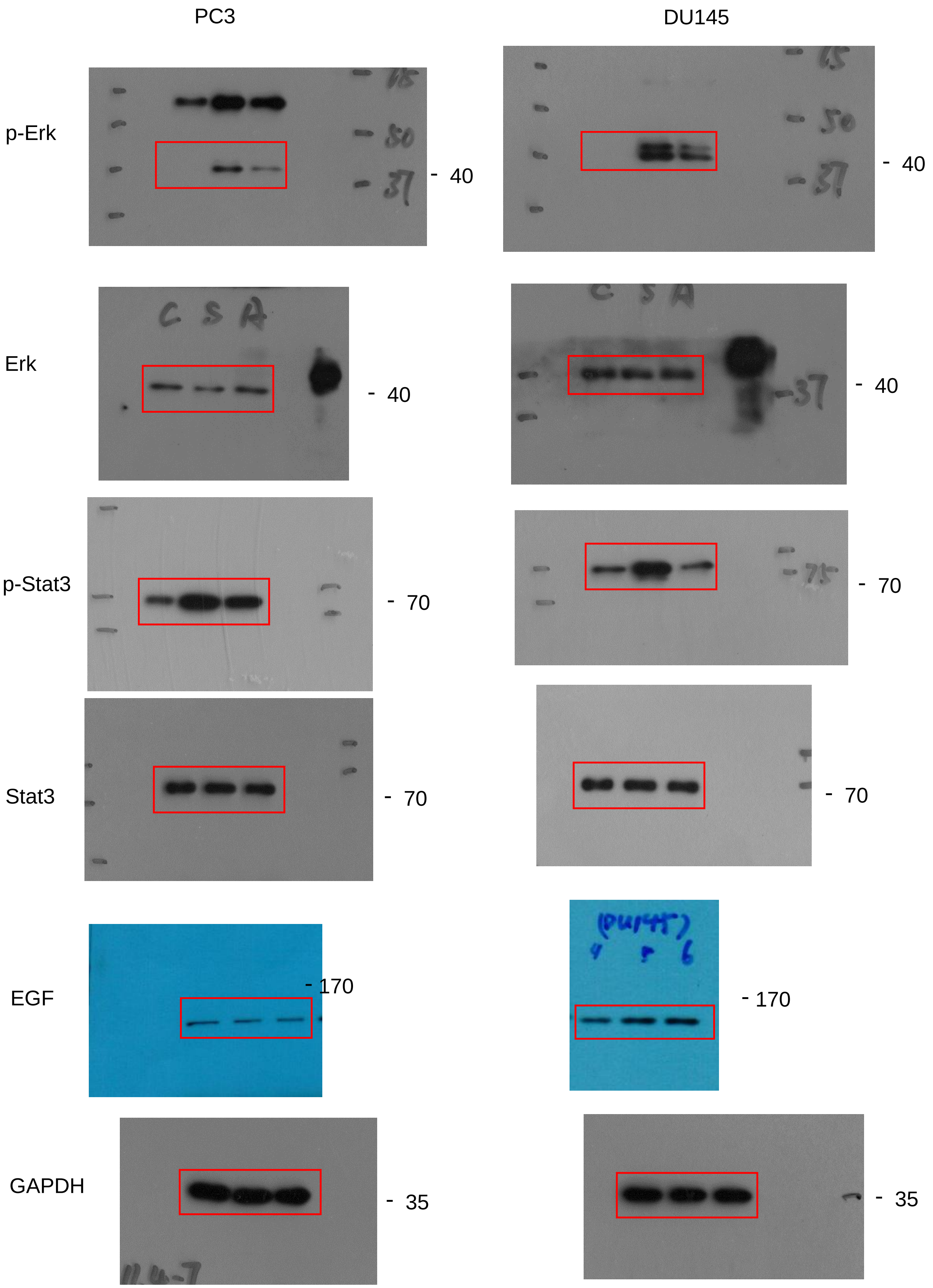

Supplementary Figure 9. The uncropped scans of immunoblots (continued)

Fig. 4b

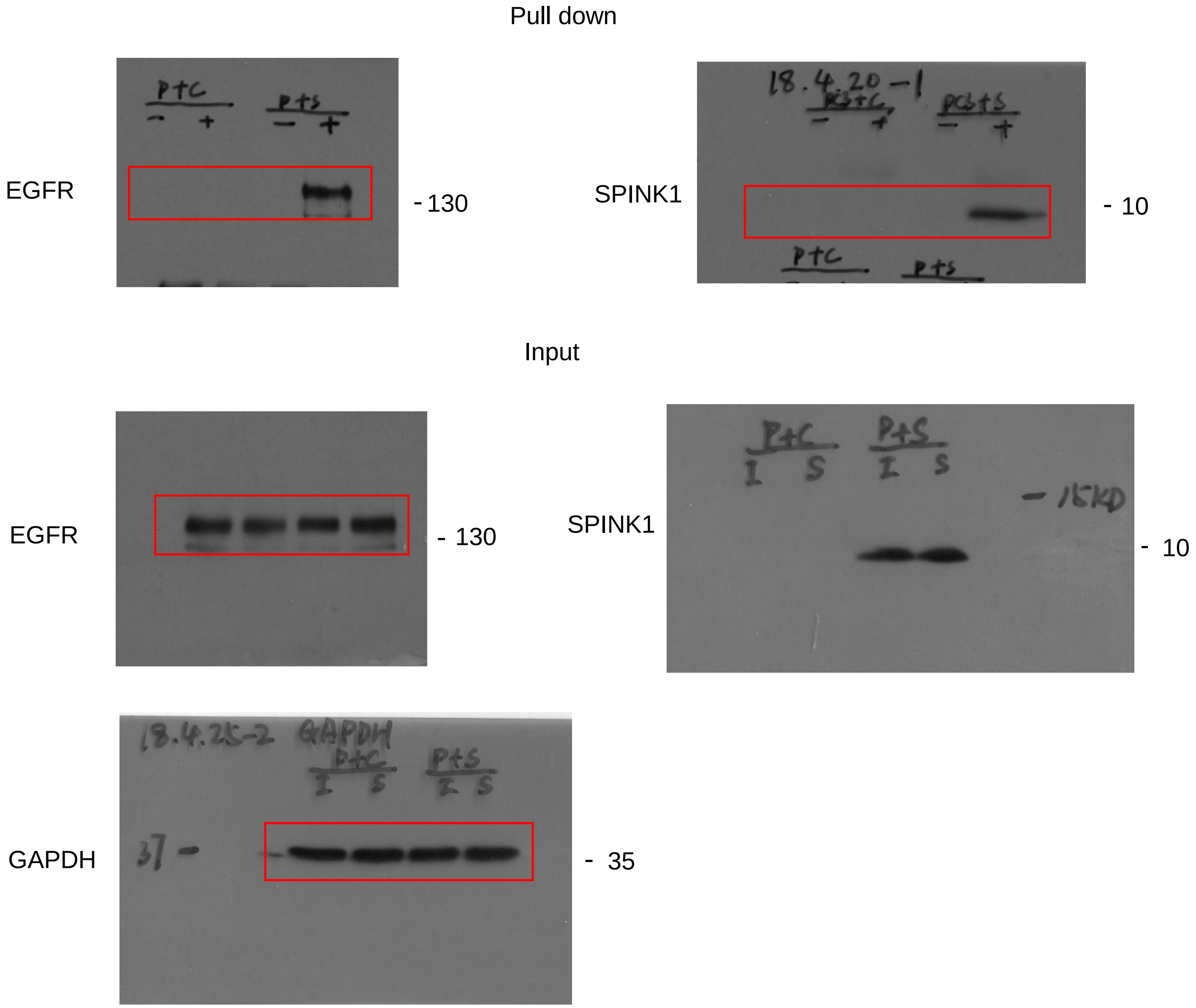

Supplementary Figure 9. The uncropped scans of immunoblots (continued)

Fig. 5h

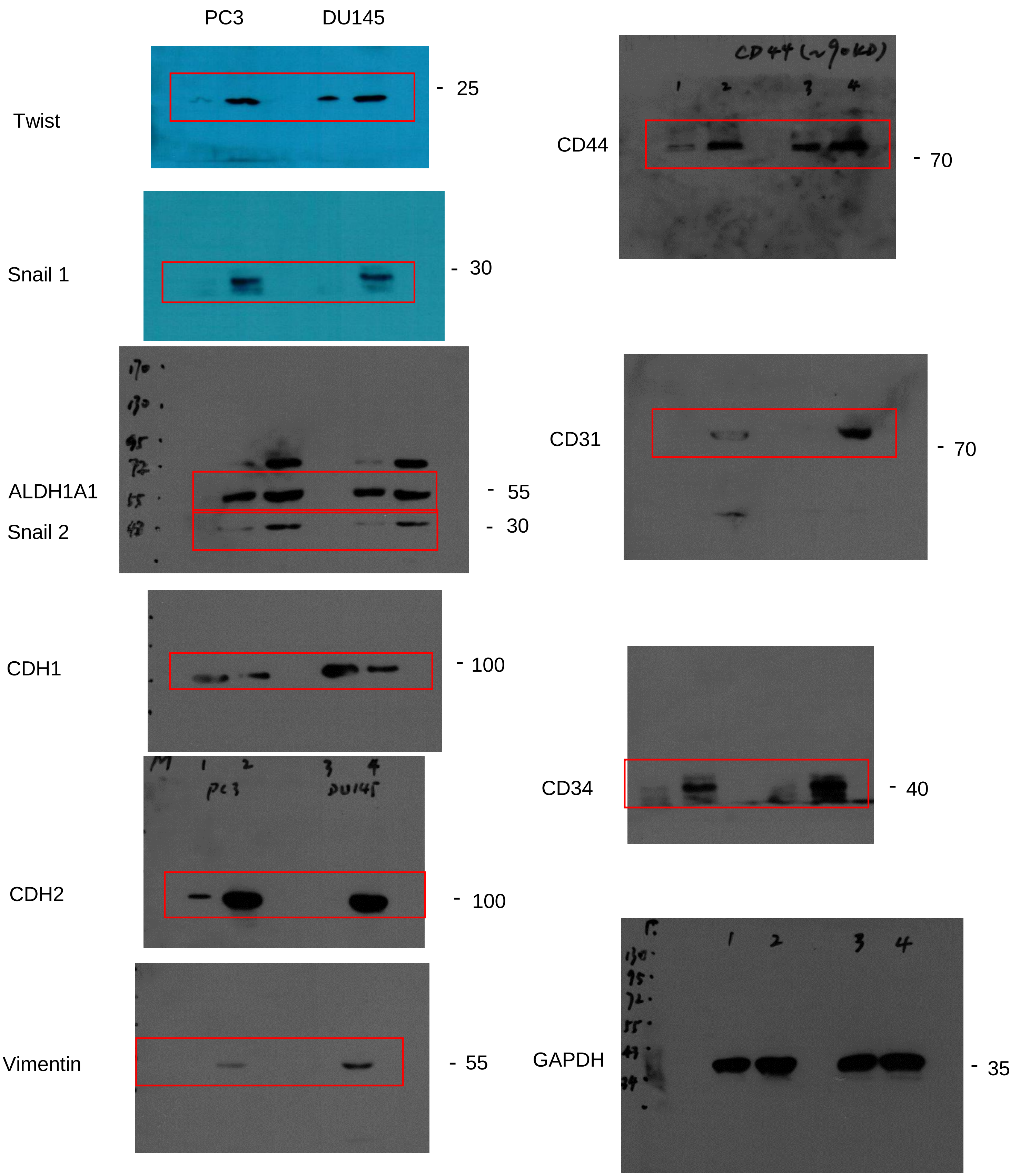

Supplementary Figure 9. The uncropped scans of immunoblots (continued)

Fig. 7d

SPINK1

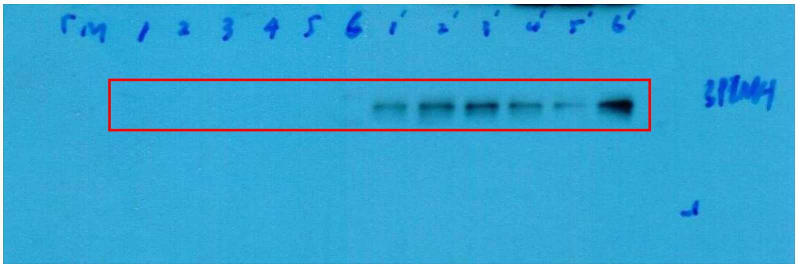

- 10

IL-8

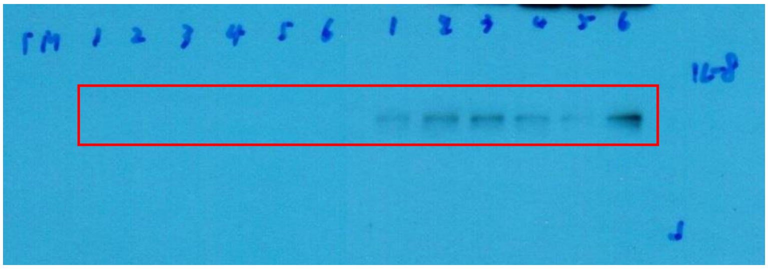

- 10

Albumin

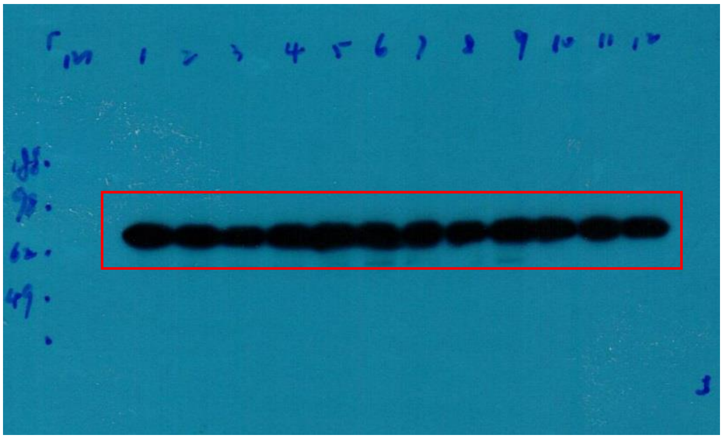

- 70

Supplementary Figure 9. The uncropped scans of immunoblots (continued)

Fig. S1f

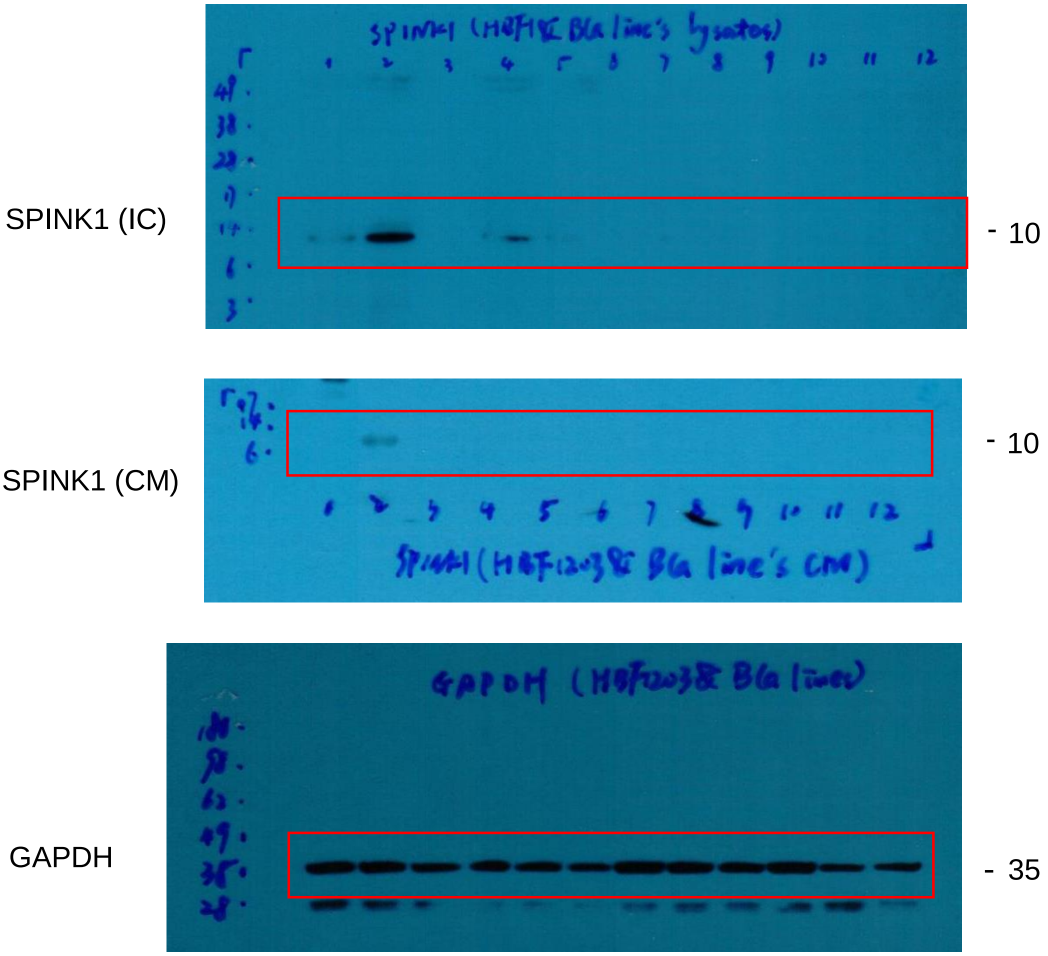

Fig. S1g

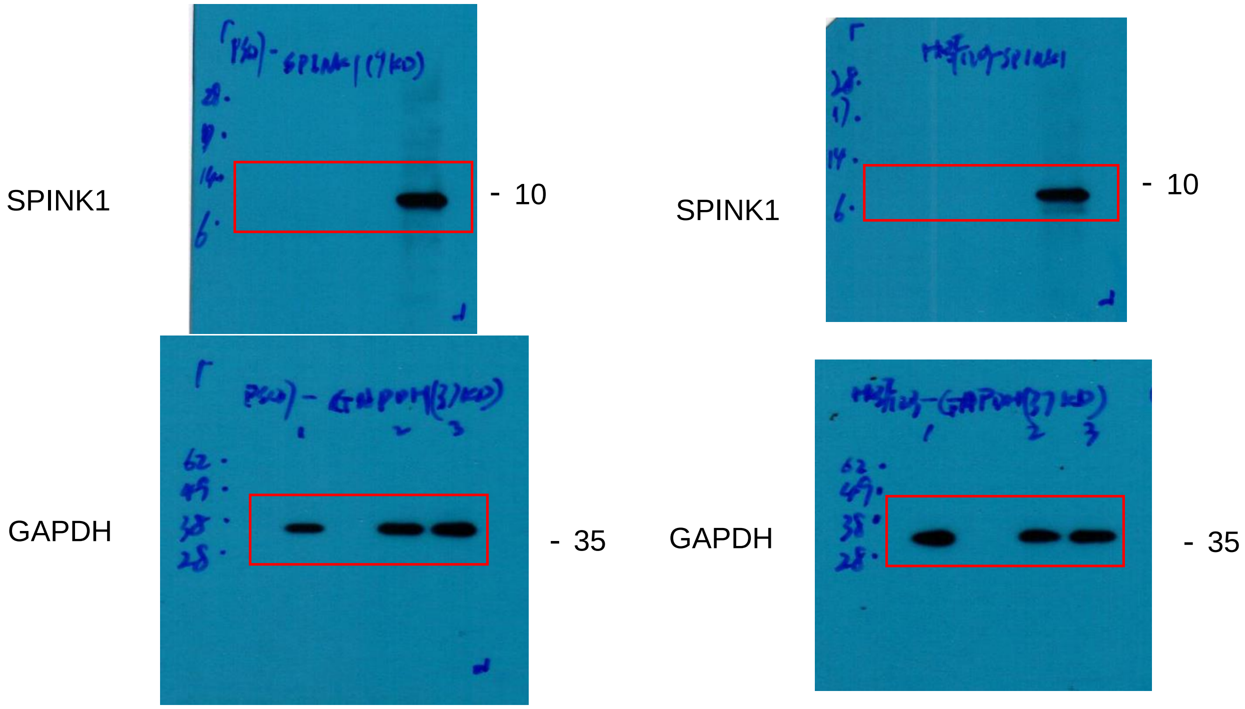

Supplementary Figure 9. The uncropped scans of immunoblots (continued)

Fig. S4a

SPINK1

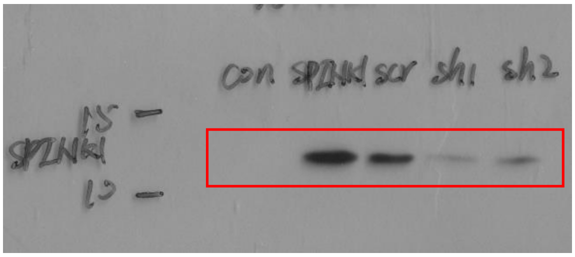

- 10

GAPDH

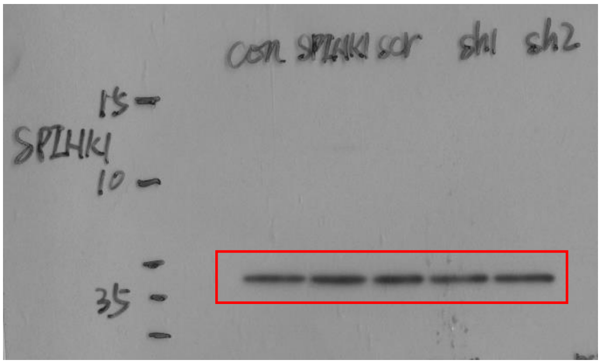

- 35

Fig. S4f

Caspase 3 (intact)

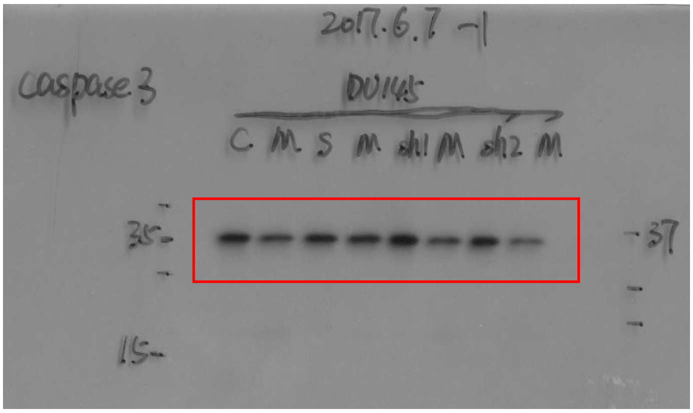

- 35

Caspase 3 (cleaved)

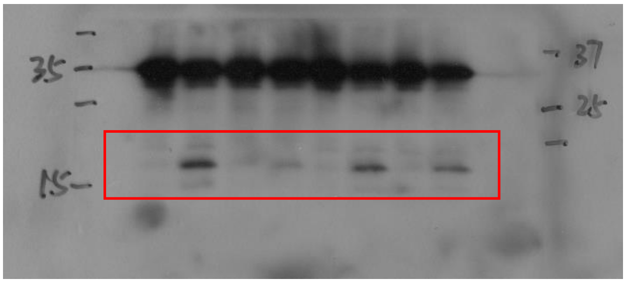

- 15

GAPDH

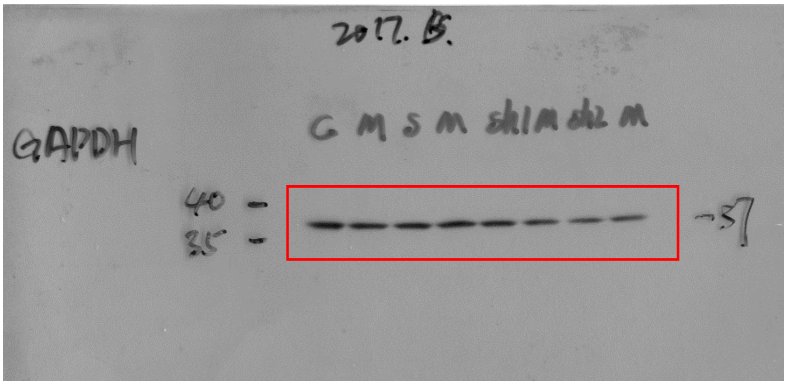

- 35

Supplementary Figure 9. The uncropped scans of immunoblots (continued)

Fig. S4j

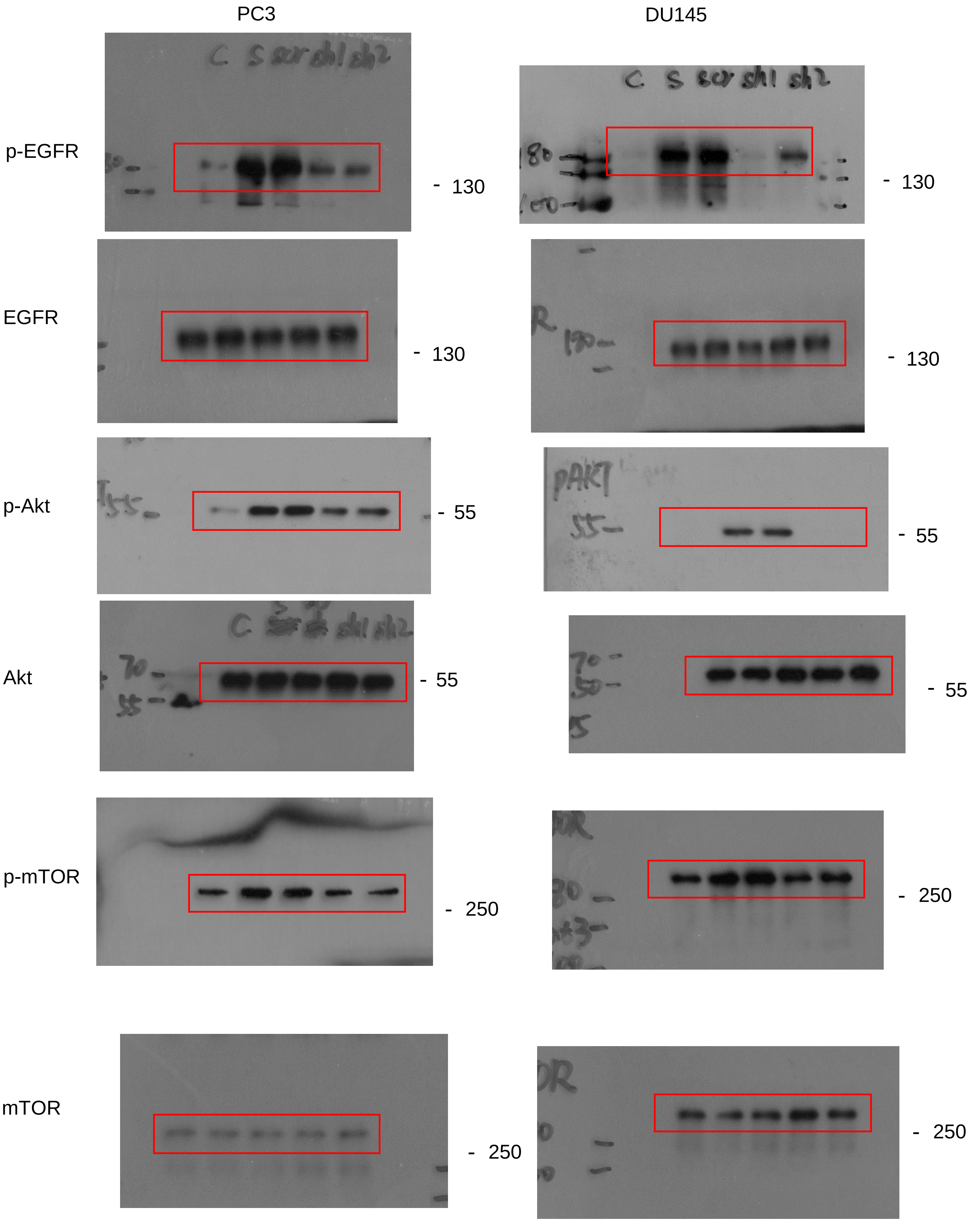

Supplementary Figure 9. The uncropped scans of immunoblots (continued)

Fig. S4j (continued)

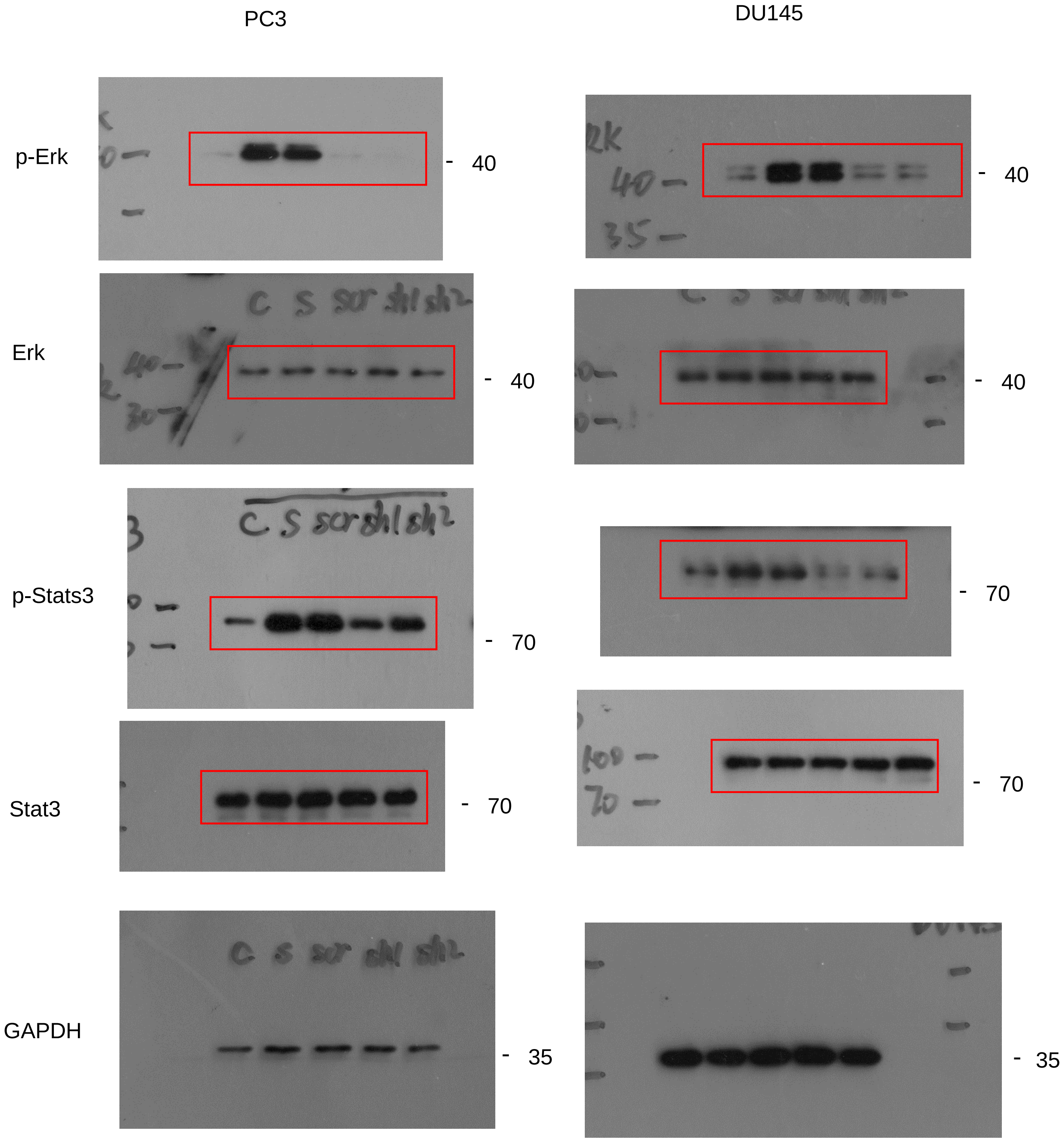

Supplementary Figure 9. The uncropped scans of immunoblots (continued)

Supplementary Table 1. Univariate and multivariate Cox proportional hazards model analysis of prognostic factors for PFS of PCa patients.

Variable	Univariate			Multivariate		
	HR	95% CI	*P*	HR	95% CI	*P*
Age: < 60 vs. ≥ 60	0.47	0.19–1.21	0.120	0.44	0.21–0.92	**0.036**
Tumor stage: IV vs. IIc-III	1.27	0.38–4.29	0.696	1.34	0.48–3.83	0.582
Tumor size: ≥ 3 vs. < 3 cm	1.52	0.67–3.44	**0.042**	2.07	1.08–4.27	**0.038**
SPINK1: low vs. high	0.74	0.33–1.65	**0.016**	0.34	0.17-1.68	**0.004**
Caspase 3 (cleaved): low vs. high	0.49	0.14–1.67	0.265	1.22	0.38–4.02	0.093
AR low vs. high	1.08	0.59-1.82	0.054	1.28	0.67-3.59	0.065

PFS, progression-free survival. PCa, prostate cancer. HR, hazard ratio. CI, confidence interval. AR, androgen receptor. *P* < 0.05 is statistically significant, with significant *P*-values highlighted in bold.

Supplementary Table 2. Univariate and multivariate Cox proportional hazards model analysis of prognostic factors for PFS of BCa patients.

Variable	Univariate			Multivariate		
	HR	95% CI	*P*	HR	95% CI	*P*
Age: < 60 vs. ≥ 60	0.39	0.15–1.04	0.096	0.42	0.19–0.82	**0.048**
Tumor stage: IV vs. Ia-III	1.39	0.32–4.02	0.508	1.30	0.38–3.65	0.498
Tumor size: ≥ 3 vs. < 3 cm	1.78	0.45–3.21	**0.046**	1.96	1.02–3.17	0.065
SPINK1: low vs. high	0.65	0.31–1.72	**0.009**	0.44	0.24-0.76	**0.004**
Caspase 3 (cleaved): low vs. high	0.67	0.25–1.89	0.228	1.56	0.39–3.22	0.106
HER2 high vs. low	2.45	1.76-3.12	**0.015**	1.85	1.23-2.79	**0.032**
PR high vs. low	2.90	0.92-3.87	0.072	1.54	0.93-2.75	0.098

PFS, progression-free survival. BCa, breast cancer. HR, hazard ratio. CI, confidence interval. HER2, human epidermal growth factor receptor 2. PR. Human progesterone *receptor*. *P* < 0.05 is statistically significant, with significant *P*-values highlighted in bold.

Supplementary Table 3. Univariate and multivariate Cox proportional hazards model analysis of prognostic factors for PFS of CRC patients.

Variable	Univariate			Multivariate		
	HR	95% CI	*P*	HR	95% CI	*P*
Age: < 60 vs. ≥ 60	1.78	0.45–3.21	0.246	1.96	1.02–3.17	0.065
Tumor stage: IV vs. II-III	1.45	0.31–4.57	0.386	1.56	0.39–2.15	0.389
Tumor size: < 3 vs. ≥ 3 cm	0.42	0.16–1.15	0.072	0.48	0.26–0.98	**0.031**
SPINK1: low vs. high	0.44	0.29–1.24	**0.048**	0.23	0.11-0.91	**0.025**
Caspase 3 (cleaved): low vs. high	0.39	0.16–1.03	0.346	1.21	0.33–2.42	0.235
EGFR low vs. high	0.737	0.324–1.602	0.189	0.760	0.382–1.491	0.167
MET low vs. high	0.821	0.319–2.059	0.231	0.840	0.390–1.863	0.175

PFS, progression-free survival. CRC, colon and rectal cancer. HR, hazard ratio. CI, confidence interval. EGFR, epidermal growth factor receptor. MET, c-MET proto-oncogene, receptor tyrosine kinase. $P < 0.05$ is statistically significant, with significant *P*-values highlighted in bold.

Supplementary Table 4. List of quantitative RT-PCR primers.

Target name	Forward (5'-3')	Reverse (5'-3')
SPINK1	CCTTGGCCCTGTTGAGTCTA	GCCCAGATTTTTGAATGAGG
IL-6	TACCCCCAGGAGAAGATTCC	TTTTCTGCCAGTGCCTCTTT
IL-8	GTGCAGTTTTGCCAAGGAGT	CTCTGCACCCAGTTTTCCTT
WNT16B	GCTCCTGTGCTGTGAAAACA	TGCATTCTCTGCCTTGTGTC
SFRP2	GCCTCGATGACCTAGACGAG	GATGCAAAGGTCGTTGTCCT
MMP1	GGTCTCTGAGGGTCAAGCAG	AGTTCATGAGCTGCAACACG
MMP3	GCAGTTTGCTCAGCCTATCC	GAGTGTCGGAGTCCAGCTTC
MMP12	ACACATTTCGCCTCTCTGCT	CCTTCAGCCAGAAGAACCTG
AREG	TGGATTGGACCTCAATGACA	AGCCAGGTATTTGTGGTTCG
GM-CSF	CCCCAGTCACCTGCTGTTAT	TGGAATCCTGAACCCACTTC
ANGPTL4	GCCTATAGCCTGCAGCTCAC	AGTACTGGCCGTTGAGGTTG
IL-1a	AATGACGCCCTCAATCAAAG	TGGGTATCTCAGGCATCTCC
IL-1b	GGGCCTCAAGGAAAAGAATC	TTCTGCTTGAGAGGTGCTGA
CXCL1	AGGGAATTCACCCCAAGAAC	TGGATTTGTCACTGTTCAGCA
CXCL3	GCAGGGAATTCACCTCAAGA	GGTGCTCCCCTTGTTCAGTA
MCP-2	TCACCTGCTGCTTTAACGTG	ATCCCTGACCCATCTCTCCT
IL-2	TGCAACTCCTGTCTTGCATT	GCCTTCTTGGGCATGTAAAA
IL-3	CTTTGCCTTTGCTGGACTTC	CCGTCCTTGATATGGATTGG
IL-5	GAGACCTTGGCACTGCTTTC	CAGTACCCCCTTGCACAGTT
IL-12	GATGGCCCTGTGCCTTAGTA	TCAAGGGAGGATTTTTGTGG
Twist 1	GTCCGCAGTCTTACGAGGAG	CCAGCTTGAGGGTCTGAATC
Twist 2	AGCAAGAAGTCGAGCGAAGA	CAGCTTGAGCGTCTGGATCT
Snail 1	TTTACCTTCCAGCAGCCCTA	CCTCATCTGACAGGGAGGTC
Snail 2	CTTTTTCTTGCCCTCACTGC	ACAGCAGCCAGATTCCTCAT
E-cadherin	TGCCCAGAAAATGAAAAAGG	GTGTATGTGGCAATGCGTTC
N-cadherin	GACAATGCCCCTCAAGTGTT	CCATTAAGCCGAGTGATGGT
Vimentin	GAGAACTTTGCCGTTGAAGC	TCCAGCAGCTTCCTGTAGGT
ALDH1A1	TGTTAGCTGATGCCGACTTG	TTCTTAGCCCGCTCAACACT
CD44	AGCAACCAAGAGGCAAGAAA	GTGTGGTTGAAATGGTGCTG
CD24	ACCCACGCAGATTTATTCCA	ACCACGAAGAGACTGGCTGT
CD31	GCAAAATGGGAAGAACCTGA	CACTCCTTCCACCAACACCT
CD34	CACCCTGTGTCTCAACATGG	GGCTTCAAGGTTGTCTCTGG
RPL13A	GTACGCTGTGAAGGCATCAA	CGCTTTTTCTTGTCGTAGGG

Supplementary Table 5. Antibodies used for Western blot (WB), immunofluorescence staining (IF) and immunohistochemistry (IHC) staining.

Antigen name	Commercial source	Catalog number (clone number）	Application	Dilution time
**SPINK1**	Abnova	H00006690-M01 (clone 4D4)	WB, IHC	1:500; 1:200
**IL-8**	Proteintech	60141-2-Ig	WB	1:500
**γH2AX**	Cell signaling	9718	IF	1:250
**BrdU**	Cell signaling	5292	IF	1:500
**EGFR**	Proteintech	18986-1-AP	WB	1:1000
**p-EGFR**	Cell signaling	2231	WB	1:1000
**mTOR**	Proteintech	20657-1-AP	WB	1:500
**p-mTOR**	Abcam	ab1093	WB	1:200
**AKT1**	Proteintech	10176-2-AP	WB	1:500
**p-AKT1**	Abcam	ab8932	WB	1:300
**Erk1/2**	Proteintech	16443-1-AP	WB	1:1000
**p-Erk1/2**	Cell signaling	4377	WB	1:500
**STAT3**	Proteintech	10253-2-AP	WB	1:1000
**p-STAT3**	Abcam	ab32143	WB	1:500
**E-cadherin**	Proteintech	20874-1-AP	WB, IF	1:500
**N-cadherin**	Proteintech	22018-1-AP	WB	1:200
**Vimentin**	Proteintech	60330-1-Ig	WB	1:1000
**Vimentin**	GeneTex	GTX100619	IF	1:500
**GAPDH**	Vazyme	Ab103	WB	1:2000
**Caspase 3 (cleaved)**	Cell signaling	9661	WB, IHC	1:500
**Caspase 3 (intact)**	Cell signaling	9662	WB	1:1000
